# Tuning Immune‐Cold Tumor by Suppressing USP10/B7‐H4 Proteolytic Axis Reinvigorates Therapeutic Efficacy of ADCs

**DOI:** 10.1002/advs.202400757

**Published:** 2024-08-29

**Authors:** Lidan Zeng, Yueming Zhu, Xin Cui, Junlong Chi, Amad Uddin, Zhuan Zhou, Xinxin Song, Mingji Dai, Massimo Cristofanilli, Kevin Kalinsky, Yong Wan

**Affiliations:** ^1^ Department of Pharmacology and Chemical Biology Emory University School of Medicine Atlanta GA 30322 USA; ^2^ Winship Cancer Institute Emory University School of Medicine Atlanta GA 30322 USA; ^3^ DGP graduate program Northwestern University Feinberg School of Medicine Chicago IL 60611 USA; ^4^ Department of Surgery UT Southwestern Medical Center Dallas TX 75390 USA; ^5^ Department of Chemistry College of Arts and Science Emory University Atlanta GA 30322 USA; ^6^ Department of Medicine Weill Cornell Medicine New York NY 10065 USA; ^7^ Department of Hematology and Medical Oncology Emory University School of Medicine Atlanta GA 30322 USA

**Keywords:** B7‐H4, deubiquitylation, tumor immunogenicity and ADC efficacy, USP10

## Abstract

Tuning immune‐cold tumor hot has largely attracted attention to improve cancer treatment, including immunotherapy and antibody‐drug conjugates (ADCs). Utilizing multiomic analyses and experimental validation, this work identifies a pivotal role for the USP10/B7‐H4 proteolytic axis in mediating the interplay between tumor immune responses and ADC efficacy, particularly for sacituzumab govitecan (SG) in treating triple negative breast cancers (TNBCs). Mechanistically, the inhibition of autocrine motility factor receptor (AMFR)‐mediated ubiquitylation of B7‐H4 by the deubiquitinase USP10 leads to the stabilization of B7‐H4, which suppresses tumor immune activity and reduces SG treatment effectiveness. Pharmacological inhibition of USP10 promotes the degradation of B7‐H4, enhancing tumor immunogenicity and consequently improving the tumor‐killing efficacy of SG. In preclinical TNBC models, suppression of USP10/B7‐H4 proteolytic axis is effective in increasing SG killing efficacy and reducing tumor growth, especially for the tumors with the USP10^high^/B7‐H7^high^ signature. Collectively, these findings uncover a novel strategy for targeting the immunosuppressive molecule B7‐H4 for cancer therapy.

## Graphical abstract

In immune‐cold triple‐negative breast cancers, elevated B7‐H4 expression diminishes immune responses and impedes the efficacy of sacituzumab govitecan. The regulation of B7‐H4 is a dynamic tug‐of‐war between degradation by E3 ligase AMFR and stabilization by deubiquitinase USP10. Inhibiting USP10 shifts the balance, restores B7‐H4 turnover, revives tumor immunogenicity, and enhances the therapeutic impact of sacituzumab govitecan.

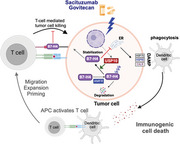

## 1 Introduction

How to tune immune‐cold tumor hot has greatly attracted our attention to improve cancer treatment.^[^ ^1^, ^2^, ^3^, ^4^ ^]^ Emergence of antibody‐drug conjugates (ADCs) shed light on a new therapeutic approach accompanied by immunotherapy in treating triple‐negative breast cancers (TNBCs).^[^ ^5^, ^6^ ^]^ TNBCs present unique therapeutic challenges due to their lack of hormone receptors and HER2 protein, which are common targets in other breast cancer subtypes.^[^ ^7^ ^]^ Although ADCs such as sacituzumab govitecan (SG) have benefited TNBC patients, certain subsets encounter therapeutic challenges due to *de novo* and acquired drug resistance.^[^ ^8^, ^9^, ^10^ ^]^ Our recent big data analyses and pathological studies with various breast cancer cohorts suggest the status of immune‐cold tumors to be a potentially critical barrier affecting the tumor response to ADC drug treatment. Nevertheless, mechanisms underlying the interplay between tumor immunogenicity and ADC drug response remain largely unclear. Our endeavor on multiomic analyses coupled with experimental validation has drawn attention to the USP10/B7‐H4 proteolytic axis as a possible pivotal target to potentiate tumor immune response and augment ADC efficacy in treating immune‐cold TNBCs.

The cytotoxic payload of ADCs is the cornerstone in the battle against tumor cells, yet its effectiveness is not solely dependent on the agents' cytotoxicity to target tumor cells.^[^ ^11^ ^]^ It also requires a conducive immune environment to optimize its therapeutic potential.^[^ ^12^, ^13^ ^]^ ADCs operate at the intersection of cancer cell targeting and immune activation, employing mechanisms like immunogenic cell death and antibody‐dependent cell‐mediated cytotoxicity, as well as initiating dendritic cell maturation. These processes not only strike directly at cancer cells but also modify the tumor microenvironment to support an immune‐mediated response. However, this efficacy can be significantly undermined in what is described as a cold‐immune environment, leading to diminished therapeutic responses.^[^ ^13^ ^]^ In this regard, understanding the interplay between ADCs and the immune environment is essential for enhancing tumor‐specific adaptive immunity that promotes a more robust and sustained immune response against tumors with ADCs treatment.

In this study, multiomic analyses identified B7‐H4 as a pivotal factor in establishing a suppressive tumor microenvironment conducive to an “immune‐cold” response. B7‐H4, also known as V‐set domain containing T‐cell activation inhibitor 1 (VTCN1), is a member of the B7 family of immune checkpoint ligands. As a transmembrane protein, B7‐H4 acts as a co‐inhibitory ligand, regulating T cells, neutrophils, and macrophages, which helps cancer cells in evading immune detection.^[^ ^14^, ^15^, ^16^, ^17^ ^]^ Notably, B7‐H4 is found not only on the cell membrane but also within the cytoplasm, where it regulates the trafficking of damage‐associated molecular patterns (DAMPs) from ER lumen to tumor cell membrane, consequently disrupting the phagocytosis of cancer cells by dendritic cells.^[^ ^3^ ^]^ Furthermore, B7‐H4 is upregulated in a range of cancers, with a notable increase especially in breast cancer.^[^ ^18^, ^19^, ^20^, ^21^ ^]^ An intriguing observation in breast cancer is the near mutual exclusivity of B7‐H4 and PD‐L1 expression, indicating that certain breast cancer cases may utilize B7‐H4 as an alternative immune checkpoint pathway.^[^ ^3^ ^]^ This dependency on B7‐H4 for immune evasion suggests that these cancers may be less responsive to anti‐PD‐L1 therapies, highlighting the need for targeted treatments against the B7‐H4 pathway.

With the reality that the immune system's engagement can be profoundly influential in ADCs combating TNBC, our protein purification coupled with mass spectrometry analysis has identified USP10 as a crucial deubiquitinating enzyme, stabilizing this immune‐suppressive protein and facilitating immune evasion that resist to ADCs‐induced tumor eradication. Interestingly, ADC treatments seem to trigger a feedback loop that boosts USP10 expression, which, in turn, increases B7‐H4 levels, further compromising the immune system's ability to monitor and attack tumor cells. This leads to diminished T cell activity, proliferation, and cytokine production. To overcome this therapeutic challenge, we have adopted a pharmacological approach to inhibit USP10, thereby diminishing B7‐H4 stability and rejuvenating immunogenicity of TNBC cells, increasing their susceptibility to the cytotoxic effects of ADCs. Our findings also reveal that suppression of USP10‐B7‐H4 proteolytic axis was effective in resensitizing SG killing efficacy and reducing tumor growth, especially for the tumors with the USP10^high^/B7‐H7^high^ signature. This study uncovers a novel strategy for targeting the immunosuppressive molecule B7‐H4 for anti‐TNBC therapy.

## 2 Results

### 2.1 Elevated B7‐H4 Expression Correlates with Immune‐Cold Feature of Breast Cancers and Poor Clinical Outcome for Antibody‐Drug Conjugate (ADC) Treatment

**Figure 1:**
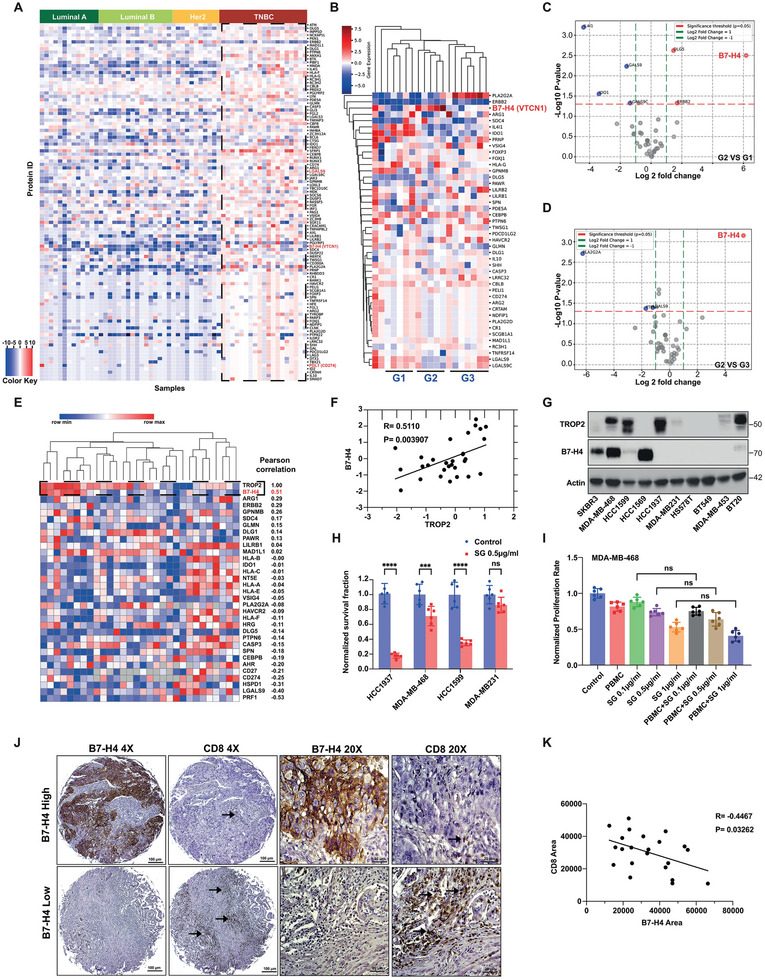
Accumulation of B7‐H4 is strongly associated with an unfavorable tumor immune response and prognosis in immune‐cold breast cancers. A) Proteomic analysis of 59 immune‐related proteins in TCGA samples of PAM50‐defined intrinsic and hormone receptor subtypes, including 19 TNBC, 13 luminal A, 17 luminal B, and 10 Her2+ breast tumors. The genes (rows) are sorted by according to protein expression difference between the average level in each subtype and the average level in the other cancer types. B) Heatmap and hierarchical clustering is based on the expression levels of the 44 immune related proteins differentially expressed in TNBC breast cancer types. Each column represents a sample; each row represents a protein. The log2 relative protein expression scale is depicted on the top left. C,D) Volcano plots showing the immune‐related protein expression changes in three clustered TNBC patient cohorts. Each circle represents one protein. The log2 fold change is represented on the x‐axis. The y‐axis shows the ‐log10 of the p value. A p value of 0.05 and a fold change of 1 are indicated by red and green lines. E,F) Heatmap and hierarchical clustering is based on the expression levels of TROP2 and other 33 immune modulating proteins‐Pearson correlation between TROP2 expression and immune related proteins were calculated (E). The correlation between TROP2 and B7‐H4 is depicted (F). G) Expression of TROP2 and B7‐H4 in various TNBC cells detected by immunoblotting. H) HCC1937, MDA‐MB‐468, HCC1599 and MDA‐MB‐231 were treated with 0.5 µg mL^−1^ SG for 48 h, clonogenic survival were determined. I) MDA‐MB‐468 cells were cocultured with human PBMCs at Effector (E) to target (T) ratio (3:1) and treated with SG for 96 h, cell proliferation rate was measured. J,K) Breast cancer specimens TMA stained for B7‐H4 and CD8 (J) and quantified staining intensity correlation between B7‐H4 and CD8 (K). ***p < 0.001, and ****p < 0.0001. Data (mean ± SEM) are representative of at least three independent experiments.

To explore novel therapeutic targets that could enhance the immunogenic cytotoxic response and overcome resistance in immune‐cold breast cancers, we undertook a comprehensive big data analysis of proteomics from The National Cancer Institute's Clinical Proteomic Tumor Analysis Consortium (CPTAC). This analysis specifically targeted over 100 proteins implicated in modulating the tumor immune microenvironment, aiming to identify pivotal molecular players that could potentiate the efficacy of existing and novel therapeutic agents.^[^ ^3^ ^]^ Our analysis revealed elevated expressions of multiple tumor immune response‐related proteins, including B7‐H4 (VTCN1), PD‐L1 (CD274), and Galectin9 (LGLAS9), in the TNBC compared to other subtypes (**Figure** **1**A). Hierarchical cluster analysis further stratifies TNBC patients into three distinct cohorts based on unique immune‐related protein expression profiles (Figure 1B). Significantly, elevated B7‐H4 expression identified via hierarchical clustering (Figure 1B), volcano plots (Figures 1C,D), and density plots (Figures S1A,B, Supporting Information) suggests a potential mechanism for immune evasion specifically leveraged by a subset of TNBCs, which may contribute to the observed reduced efficacy of immunotherapies and further hamper the effectiveness of cytotoxic agents’ combinational strategies in patients with high B7‐H4 expression. This relationship is further substantiated by the informatic analysis in Figure S1C, Supporting Information, which indicates a negative correlation between elevated B7‐H4 levels and the induction of endoplasmic reticulum stress, preventing the release of DAMPs in TNBC patients.

We also investigated whether increased B7‐H4 protein levels affect the cytotoxic efficacy of ADCs such as SG, which depends on a supportive immune environment for optimal and sustained therapeutic effect. SG is a novel antibody‐drug conjugate composed of SN‐38, a potent topoisomerase I inhibitor, linked to an anti‐TROP‐2 monoclonal antibody. Our analysis found that TNBC patients in CPTAC cohorts with high TROP2 expression exhibited a significant positive correlation with B7‐H4 expression (Figure 1E,F), indicating that patients eligible for SG treatment often have an immunosuppressive environment. Additionally, we evaluated the baseline expression levels of TROP2 and B7‐H4 in various TNBC cell lines, including HCC1937 (high TROP2, low B7‐H4), MDA‐MB‐468 (high TROP2, high B7‐H4), HCC1599 (high TROP2, low B7‐H4), and MDA‐MB‐231 (low TROP2, low B7‐H4) (Figure 1G). Based on clonogenic survival assays, we observed that among the cell lines with high TROP2, MDA‐MB‐468 cells with high B7‐H4 expression levels showed markedly reduced sensitivity to SG treatment in comparison with HCC1599 cells and HCC1937 cells with low B7‐H4 (Figure 1H, Figure S1D, Supporting Information). To investigate whether a B7‐H4‐enriched immunosuppressive environment contributes to resistance against SG efficacy, we treated MDA‐MB‐468 and HCC1937 cells co‐cultured with peripheral blood mononuclear cells (PBMCs) with varying doses of SG. The results in Figure 1I and Figure S1E, Supporting Information indicated that, while PBMC co‐culture did not enhance SG‐mediated cytotoxicity in B7‐H4‐high MDA‐MB‐468 cells (Figure 1I), it significantly potentiated tumor cell eradication in HCC1937 cells (Figure S1E, Supporting Information). To confirm the observations in Figure 1A–I and determine if the increased expression of B7‐H4 can function as a co‐inhibitory ligand to inhibit T‐cell function, which in turn facilitates tumor evasion and lowers immunogenic cell death, we performed the T cell infiltration correlation analysis and ssGSEA pathway analysis. Figure S1F,G, Supporting Information demonstrated that increased B7‐H4 expression is associated with decreased CD8+ and CD4+ T cell infiltration in TNBCs. Additionally, the ssGSEA MsigDB‐based pathway analysis confirmed that high B7‐H4 expression correlates positively with decreased T cell‐mediated immunity, cytokine production, and immune effector processes (Figure S1H–P, Supporting Information). Next, we analyzed a tissue microarray containing 110 unique breast tissue samples via immunohistochemistry. In most TNBC samples, we observed a significant increase in B7‐H4 protein expression coinciding with a reduction in the CD8+ T cell population (Figure 1J,K), further confirming the effect of high B7‐H4 levels in enhancing immune evasion. In addition, high B7‐H4 expression is linked with lower overall survival as compared to other BC cancer patients using TCGA database analysis (Figure S1Q, Supporting Information).^[^ ^22^ ^]^ These results collectively establish a significant association between elevated B7‐H4 expression and poorer clinical outcomes in TNBC patients potentially receiving SG therapy, highlighting the importance of B7‐H4 as a potential therapeutic target to enhance the immune system's effectiveness with ADC treatment.

### 2.2 Identification of USP10 as a Deubiquitinase that Stabilizes B7‐H4 through Counteracting B7‐H4 Ubiquitylation in Immune‐Evasive Triple Negative Breast Cancers (TNBCs)

**Figure 2:**
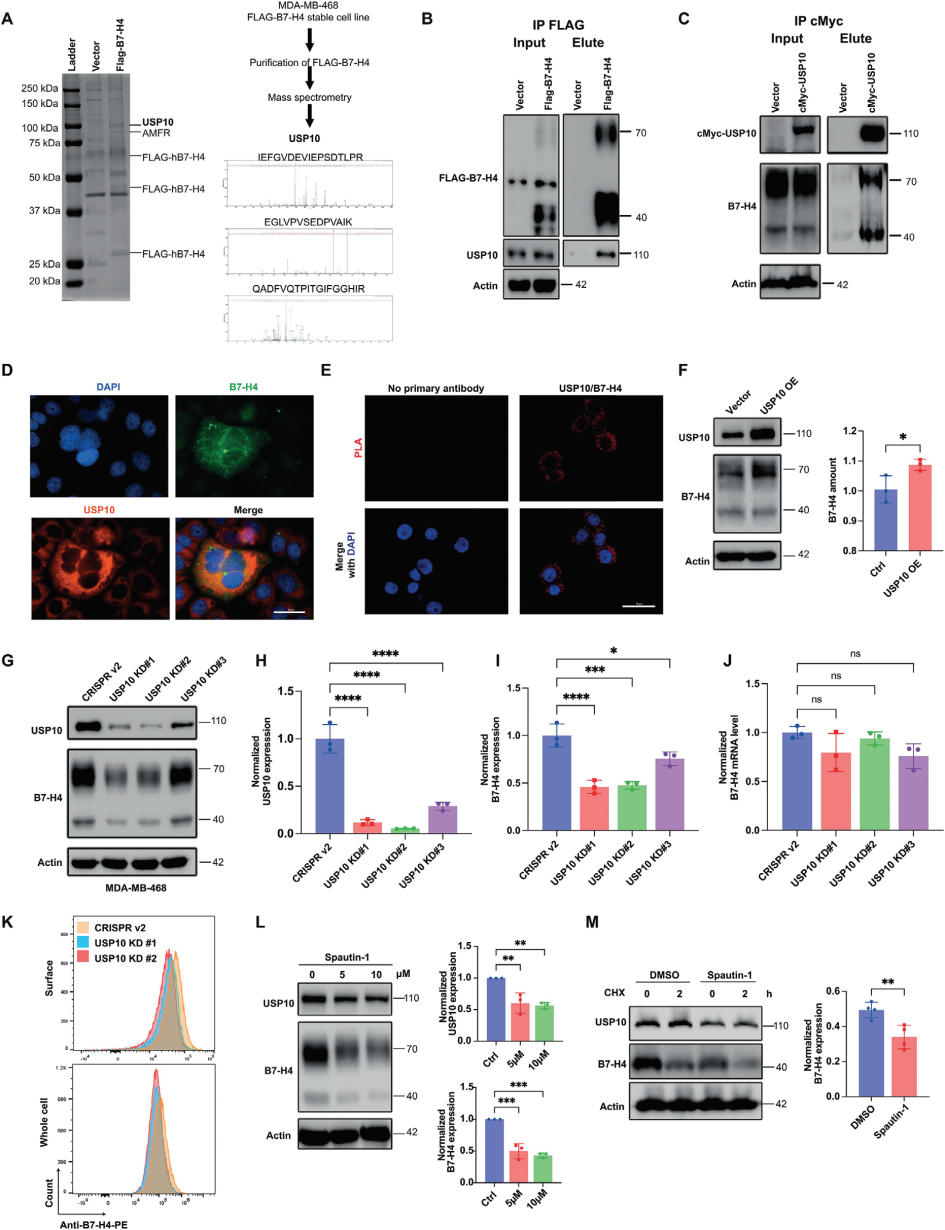
Identification of USP10 as a binding partner of B7‐H4 that upregulates B7‐H4 protein levels in immune‐cold TNBC breast tumors. A) Coomassie blue staining of B7‐H4 immunoprecipitated from MDA‐MB‐468 cells expressing FLAG‐tagged B7‐H4 reveals USP10 as a binding partner by mass spectrometry analysis. B,C) Confirmation of the B7‐H4/USP10 interaction via co‐immunoprecipitation by coimmunoprecipitation of endogenous USP10 (B) and coimmunoprecipitation of endogenous B7‐H4 (C). D) Immunofluorescence staining of MDA‐MB‐468 cells expressing FLAG‐B7‐H4 shows colocalization with USP10. Scale bar = 30 µm. E) MDA‐MB‐468‐FLAG‐B7‐H4 cells were subjected to Duolink in Situ PLA assay with FLAG mouse antibody and USP10 rabbit antibody. Red dots indicate the interaction of the two proteins. Scale bar = 30 µm. F) Overexpression of USP10 in MDA‐MB‐468 cells enhances B7‐H4 protein levels, evidenced by immunoblotting (left panel) and subsequent quantification (right panel). G–J) USP10 knockdown results in downregulation of B7‐H4 protein but not mRNA in MDA‐MB‐468 cells. Three USP10 knockdown cell lines were established with different sgRNAs with CRISPR/Cas9 system. Empty vector CRISPRv2 used as the control. (G) Representative images of immunoblotting of USP10 and B7‐H4. (H) Quantification of USP10 and (I) B7‐H4 protein normalized with β‐actin amount in biological triplicates. (J) Quantification of B7‐H4 mRNA levels with qPCR. K) Decreased membrane and total B7‐H4 in USP10 knockdown cells. MDA‐MB‐468 cells were stained with PE conjugated anti‐human B7‐H4 antibody without or with permeabilization for surface B7‐H4 and total B7‐H4, respectively and subjected to flow cytometry analysis. Representative images are shown. L) Inhibition of USP10 with spautin‐1 leads to decreased B7‐H4 protein in MDA‐MB‐468 cells, validated by western blot (left panel) and quantified intensity (right panel). M) Spautin‐1 treatment increases B7‐H4 turnover. MDA‐MB‐468 cells were treated with DMSO or 10 µM spautin‐1 for 24 h and then treated with 100 µg mL^−1^ cycloheximide (CHX) at the indicated time points. Representative images of immunoblotting of B7‐H4 (left panel). Quantification of B7‐H4 (40 kDa) intensity normalized to time 0 (right panel). *p < 0.05, **p < 0.01, ***p < 0.001, and ****p < 0.0001. Data (mean ± SEM) are representative of at least three independent experiments.

To identify the mechanism that governs B7‐H4 protein turnover and stability in immune‐cold TNBCs, we employed Tandem Affinity Purification (TAP) and mass spectrometry to analyze the B7‐H4 interactome in MDA‐MB‐468 cells stably expressing FLAG‐B7‐H4. The Flag‐tagged B7‐H4 protein was overexpressed, affinity‐purified using an empty vector as a control and subjected to mass spectrometry.^[^ ^3^ ^]^ This led to identification of USP10 as a key regulator potentially responsible for the elevated levels of B7‐H4 in TNBC. Concurrently, AMFR was identified as the ubiquitin E3 ligase that regulates B7‐H4 for ubiquitylation and subsequent proteasomal degradation (**Figure** **2**A). To characterize the biochemical implications of the USP10/B7‐H4 interaction and its potential physiological significance, we conducted a series of co‐immunoprecipitation assays in MDA‐MB‐468 cells. Results, as shown in Figure 2B,C, demonstrated a co‐interaction wherein endogenous USP10 was co‐immunoprecipitated with FLAG‐tagged B7‐H4, and conversely, endogenous B7‐H4 was present in the c‐Myc‐tagged USP10 immune complexes. To determine the subcellular localization of this interaction, the specific intracellular interaction regions between B7‐H4 and USP10 were further elucidated using immunofluorescence staining (Figure 2D) and validated by a Duolink proximity ligation assay (Figure 2E). The results confirmed that the B7‐H4 and USP10 interaction occurs within the cytosolic compartment. To examine the direct role of USP10 on B7‐H4 turnover via deubiquitinase activity, MDA‐MB‐468 human breast cancer cells were genetically modified to stably overexpress or knockdown USP10. Figure 2F illustrates that enhanced USP10 levels corresponded with an increase in B7‐H4 protein levels. Similar increases were also observed in additional cell lines such as SKBR3 and HEK‐293T (Figure S2A,B, Supporting Information). Conversely, CRISPR/Cas9‐mediated knockdown of USP10 resulted in a reduction in B7‐H4 protein expression but not mRNA level across both MDA‐MB‐468 cells (Figure 2G–J) and SKBR3 breast cancer cells (Figure S2C, Supporting Information), further confirming the regulatory role of USP10 in counteracting B7‐H4 ubiquitylation resulting in B7‐H4 stabilization. To investigate whether USP10 mediates the cytosolic deubiquitylation of B7‐H4 and its subsequent translocation to the plasma membrane, we utilized flow cytometry with a fluorophore conjugated anti‐B7‐H4 antibody to measure B7‐H4 presence on the surface of USP10 knockdown (KD) MDA‐MB‐468 cells. The fluorescence intensity of cell membrane‐bound B7‐H4 was notably diminished in USP10‐deficient MDA‐MB‐468 cells (Figure 2K) compared to the control, with analogous observations in SKBR3 cells (Figure S2D, Supporting Information). Moreover, treatment with spautin‐1, an established USP10 inhibitor, resulted in a decrease in B7‐H4 protein levels (Figure 2L and Figure S2E, Supporting Information) and increased B7‐H4 turnover (Figure 2M), underscoring the potential of pharmacological inhibition of USP10 as a strategy to modulate B7‐H4 levels. Given that spautin‐1 was initially identified as an inhibitor of both USP10 and USP13 with similar IC50 values,^[^ ^23^ ^]^ we evaluated whether spautin‐1‐induced B7‐H4 downregulation could also occur through USP13 inhibition. Treatment of MDA‐MB‐468 cells with spautin‐1 for 48 h significantly reduced USP10 and B7‐H4 protein levels but did not affect USP13 protein levels (Figure S2F–I, Supporting Information). Additionally, USP13 KD in MDA‐MB‐468 cells did not alter B7‐H4 protein expression (Figure S2J–M, Supporting Information). To further validate our findings, we used Wu‐5, another specific USP10 inhibitor,^[^ ^24^ ^]^ which also resulted in a noticeable reduction in USP10 and B7‐H4 (Figure S2N–Q, Supporting Information). These results collectively underscore the specific role of USP10 in regulating B7‐H4 expression. Subsequent MsigDB database pathway analysis also observed the elevation of B7‐H4 expression in TNBC cohorts, which appears to be closely associated with an upregulated deubiquitylation pathway (Figure S3A,B, Supporting Information). Notably, this analysis paralleled findings in Figure 1B, identifying a subgroup within TNBC with concurrent upregulation of USP10 and B7‐H4 protein levels (Figure S3C, Supporting Information) and reinforcing the potential interplay between USP10 activity and B7‐H4 stability in this breast cancer subset.

**Figure 3:**
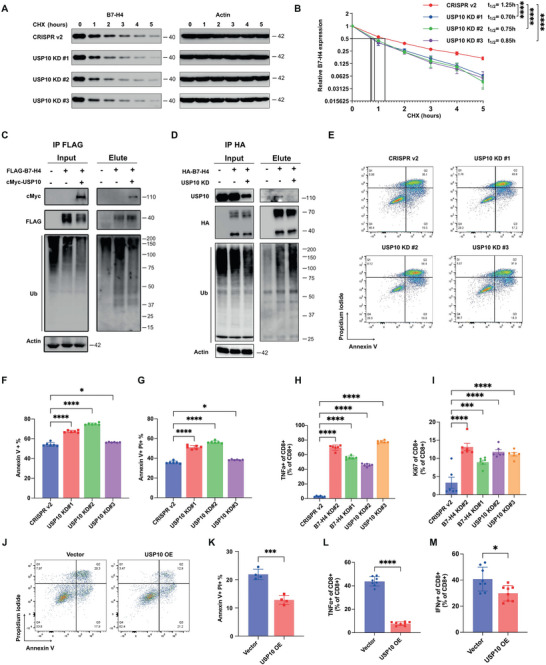
USP10 is a pivotal regulator to stabilize B7‐H4 via deubiquitylation and modulate T cell function. A,B) USP10 knockdown leads to increased B7‐H4 turnover in MDA‐MB‐468. A) Pulse‐chase analysis for the control CRISPR v2 and USP10 knockdown MDA‐MB‐468 cells with 100 µg mL^−1^ cycloheximide. B) Quantification of the amount of B7‐H4 (40 kDa) normalized to the initial time point. The half‐life t_1/2_ is estimated based on the plot. C) USP10 overexpression reduces B7‐H4 ubiquitylation in HEK‐293T stable cell line expressing FLAG‐B7‐H4. FLAG‐B7‐H4 was immunoprecipitated followed by immunoblotting with anti‐ubiquitin. D) USP10 knockdown enhances B7‐H4 ubiquitylation in MDA‐MB‐468 cells. USP10 was knocked down in MDA‐MB‐468 cells stably expressing HA‐tagged B7‐H4. Following immunoprecipitation of HA‐B7‐H4, ubiquitylation levels were assessed via immunoblotting with an anti‐ubiquitin antibody. E–G) Flow cytometry analysis of MDA‐MB‐468 cells cocultured with PBMCs shows increased apoptosis in USP10 knockdown cells (E). Quantitative analysis of the percentage of total apoptosis (Annexin V+) (F) and late apoptosis (Annexin V+ and PI+) cancer cells (G). H,I) USP10 knockdown or B7‐H4 knockdown in MDA‐MB‐468 releases the suppression of the function of cytotoxic T cells by cancer cells as determined by the percentage of TNFα positive cytotoxic T cells (H) and the percentage of Ki67+ cytotoxic T cells (I). J,K) Flow cytometry analysis of MDA‐MB‐468 cells cocultured with PBMCs shows decreased apoptosis in USP10 overexpression cells (J). Quantitative analysis of the percentage of late apoptosis (Annexin V+ and PI+) cancer cells (K). L,M) USP10 overexpression in MDA‐MB‐468 further suppressed the function of cytotoxic T cells by cancer cells as determined by the percentage of TNFα+cytotoxic T cells (L) and the percentage of IFN‐γ+ cytotoxic T cells (M). *p < 0.05, **p < 0.01, ***p < 0.001, and ****p < 0.0001 by the one‐way ANOVA test or two‐way ANOVA test, Data (mean ± SEM) are representative of at least three independent experiments.

To determine if USP10 directly affects B7‐H4 turnover dynamics, we conducted a pulse‐chase assay in MDA‐MB‐468 cells with CRISPR/Cas9‐mediated USP10 knockdown. Our results showed that USP10 depletion significantly accelerated B7‐H4 protein turnover (**Figure** **3**A,B), reducing the B7‐H4 protein half‐life from 1.25 to 0.70–0.85 h. To examine the role of USP10 on B7‐H4 degradation through catalyzing B7‐H4 deubiquitylation, ubiquitylation assays were conducted in HEK293T‐FLAG‐B7‐H4 cells with enforced USP10 expression. Immunoprecipitation of endogenous B7‐H4 followed by subsequent immunoblotting for ubiquitin revealed that USP10 overexpression attenuates B7‐H4 ubiquitylation (Figure 3C). Conversely, knocking down USP10 or treatment of spautin‐1 resulted in an appreciable increase in B7‐H4 ubiquitylation, reinforcing the role of USP10 in the preservation of B7‐H4 protein levels (Figure 3D and Figure S3D, Supporting Information). To further confirm the specific role of USP10 in regulating B7‐H4 turnover and to determine if other USPs may exhibit similar regulatory effects in a TNBC setting, we aligned the protein sequences of all human USPs and identified those with high sequence similarity to USP10. Based on the phylogenetic analysis, we identified 10 USPs closely related to USP10: USP36, USP17, USP42, USP30, USP49, USP44, USP3, USP28, USP25 (Figure S4A, Supporting Information). We designed sgRNAs to knock down these USPs and examined B7‐H4 expression in breast cancer cells. None of these USPs significantly reduced B7‐H4 levels (Figure S4B,C, Supporting Information). Additionally, results from a cycloheximide (CHX)‐based chase assay demonstrated that other USPs do not affect B7‐H4 protein turnover (Figure S4D,E, Supporting Information). To investigate the potential role of USP10 in facilitating immune escape by cancer cells, we utilized a co‐culture system with cancer cells and human PBMCs, activating cytotoxic T‐cells with anti‐human CD3 and CD28.2 antibodies. Following co‐culture, cancer cells were assessed for apoptosis using Annexin V/Propidium Iodide staining and analyzed via flow cytometry. Results revealed a significant increase in apoptotic cell count in USP10 knockdown populations compared to controls, indicating that loss of USP10 sensitizes cancer cells to T‐cell mediated cytotoxicity (Figure 3E–G). In contrast, USP10 knockdown did not induce apoptosis in the absence of PBMCs (Figure S4F–H, Supporting Information). Furthermore, the depletion of USP10 was associated with enhanced cytotoxic T‐cell activity, as evidenced by increased Ki67 expression, indicating higher T‐cell proliferation, and elevated TNFα+ CD8+ T cell population (Figure 3H,I). Conversely, overexpression of USP10 in MDA‐MB‐468 cells led to a marked reduction of apoptosis accompanied with decreased TNFα+ and IFN‐γ+ CD8+ T cell population (Figure 3J–M and Figure S4I–K, Supporting Information). These findings collectively suggest that the deubiquitinase USP10 is instrumental in maintaining high levels of B7‐H4, which in turn promotes an immune‐suppressive microenvironment by impairing T‐cell efficacy in immune‐cold TNBC settings.

### 2.3 Mapping and Structural Modeling of the Interaction between USP10 and B7‐H4

**Figure 4:**
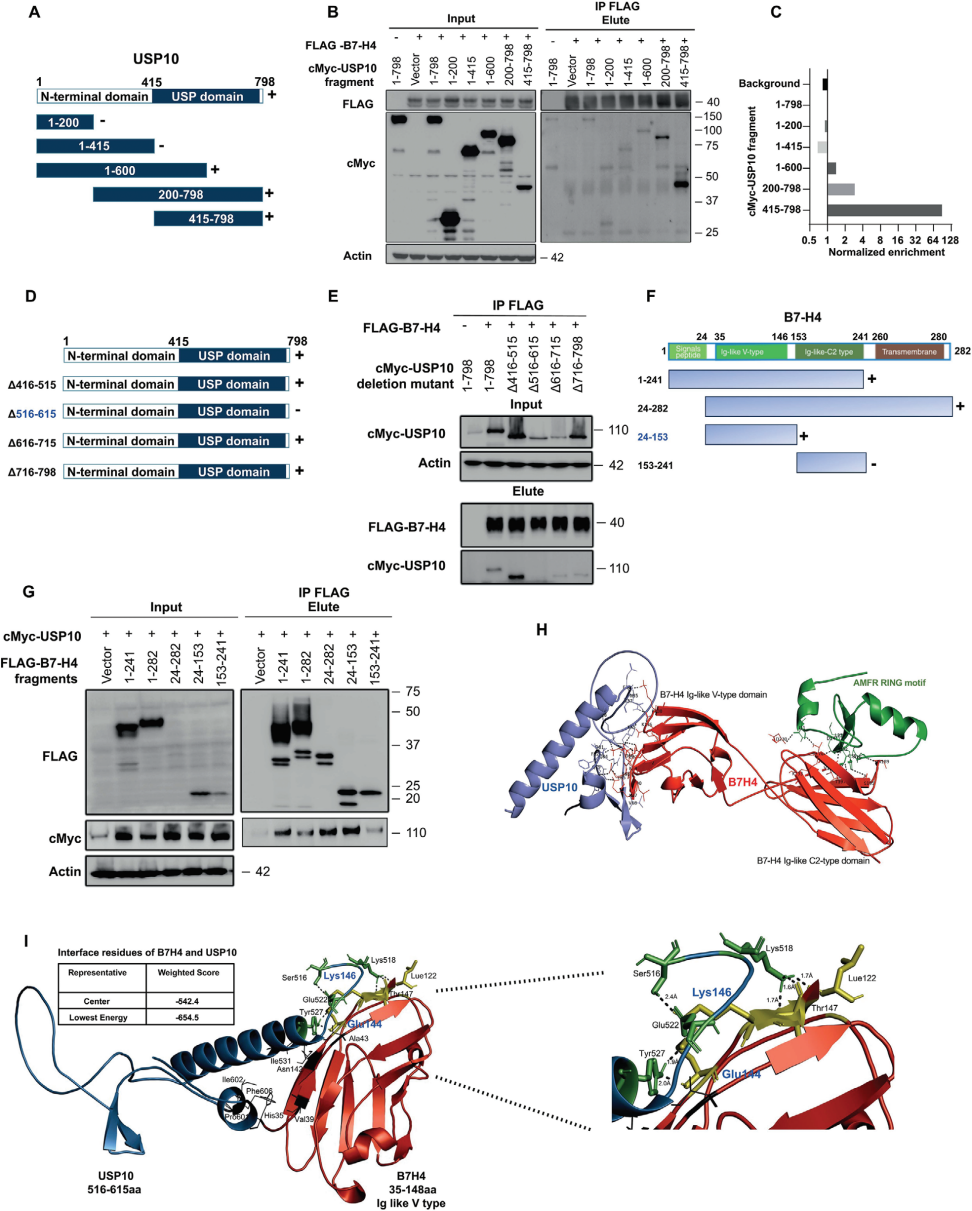
Molecular mapping the domains on USP10 and B4‐H4 that facilitates the interaction between USP10 and B7‐H4. A–E) Schematic diagram of human USP10 domains and strategy to engineer a series of USP10 fragments (A) and USP10 deletion mutants (D). Mapping of the molecular domain on USP10 involving in the interaction with B7‐H4. The interactions between B7‐H4 and USP10 fragments were examined by co‐IP experiments in HEK‐293T cells. Amino acid stretches from 516 to 615 amino acid on USP10 (B‐E) were identified as the region that mediates its interaction with B7‐H4. F) Schematic diagram of human B7‐H4 domains and strategy to engineer a series of B7‐H4 deletion fragments. G) The interactions between USP10 and B7‐H4 fragments were examined by co‐IP experiments in HEK‐293T cells. Amino acid stretches from 24 to 146 (including Ig like V‐type domain) on B7‐H4 were identified as the region that mediates the interaction between USP10 and B7‐H4. H) Molecular docking model of the interaction between USP10‐B7‐H4‐AMFR complex. AMFR is the E3 ligase for B7‐H4. The ring motif of AMFR is in green; the Ig‐like V‐type and Ig‐like‐C2 type domain of B7‐H4 is in red and 516–615 residues of USP10 is in blue. The docking model shows that USP10 binds to the Ig‐like V‐type domain of B7‐H4 and the deubiquitinase AMFR binds to the Ig‐like‐C2 type domain of B7‐H4, where the interacting residues are highlighted in red and green line. I) The Structural model for the interaction of USP10 (sky blue) with B7‐H4 (red) investigated through docking studies (left panel). Docking simulations have explored the potential binding sites and energetics of the B7‐H4‐USP10 interaction with lowest energy −654.5 kJ mol^−1^, and also revealed amino acid residues (Lue122, Glu144 Lys146 and Thr147) of B7‐H4 (yellow) that forming conventional hydrogen bond interaction with USP10 (green) residue (Lys518, Ser516, Glu522, Tyr527) and further stabilized by other hydrophobic interactions between (Ile531, Pro601, Ile602, Phe606) and (His35, Val39, Ala43, Asn142), respectively. The zoomed view of interface indicating the close interaction between B7‐H4 (stick yellow) with USP10 (stick green) shows in (right panel).

To uncover the mechanism by which B7‐H4 is recognized by USP10 allowing the removal of its attached ubiquitin‐conjugates, we engineered a set of B7‐H4 deletion fragments with Flag tag and fragments of USP10 with c‐Myc tag, which were then co‐expressed in HEK‐293T cells for subsequent co‐immunoprecipitation (co‐IP) analyses. We crafted and expressed a suite of c‐Myc‐tagged USP10 fragments (**Figure** **4**A) alongside Flag‐tagged B7‐H4 in HEK‐293T cells. The resulting co‐IP assays pinpointed the USP domain stretching from amino residue 415–798 at the C‐terminus of USP10 as integral for binding B7‐H4 (Figure 4B,C). To further identify the exact site responsible for USP10‐B7‐H4 interaction, a series of USP domain deletion mutants were engineered, subsequently co‐IP assay demonstrated that the mid‐segment of the USP domain (amino acid 516–615) emerged as key in mediating this interaction, as its removal negated the binding (Figure 4D,E). To identify the interacting domain on B7‐H4, different B7‐H4 fragments were co‐transfected with Flag‐tagged USP10 in HEK‐293T cells. The co‐IP assay indicated the Ig‐like V‐type domain of B7‐H4 as the primary site for USP10 interaction (Figure 4F,G), with amino acid residues 24–153 of B7‐H4 being essential for their interplay. We have recently explored and characterized the role of AMFR acting as an E3 ligase responsible for the ubiquitin‐proteasomal degradation of B7‐H4. To comprehensively address the mechanism by which the interplay of AMFR‐mediated ubiquitylation and USP10‐guided deubiquitylation in regulating B7‐H4 proteolysis, we conducted structural modeling of the interaction between B7‐H4, AMFR, and USP10 by integrated docking simulations. We generated in silico structural models for the complexes B7‐H4‐AMFR and B7‐H4‐USP10, consistent with experimental data for the interfaces formed between B7‐H4 and the two respective enzymes AMFR and USP10 (Figure 4H). Subsequently, we engaged in comprehensive molecular docking simulations to generate molecular models of the interaction between amino acids 516–615 on USP10 and B7‐H4's Ig‐like V‐type domain. Docking simulations have explored the potential binding sites and energetics of the B7‐H4‐USP10 interaction with lowest energy −654.5 kJ mol^−1^, and revealed amino acid residues (Lue122, Glu144 Lys146 and Thr147) of B7‐H4 (yellow) that forming covalent interaction with USP10 (green) residue (Lys518, Ser516, Glu522, Tyr527). The interface of B7‐H4‐USP10 is further stabilized by other hydrophobic interactions between (His35, Val39, Ala43, Asn142) and (Ile531, Pro601, Ile602, Phe606), respectively (Figure 4I).

**Figure 5:**
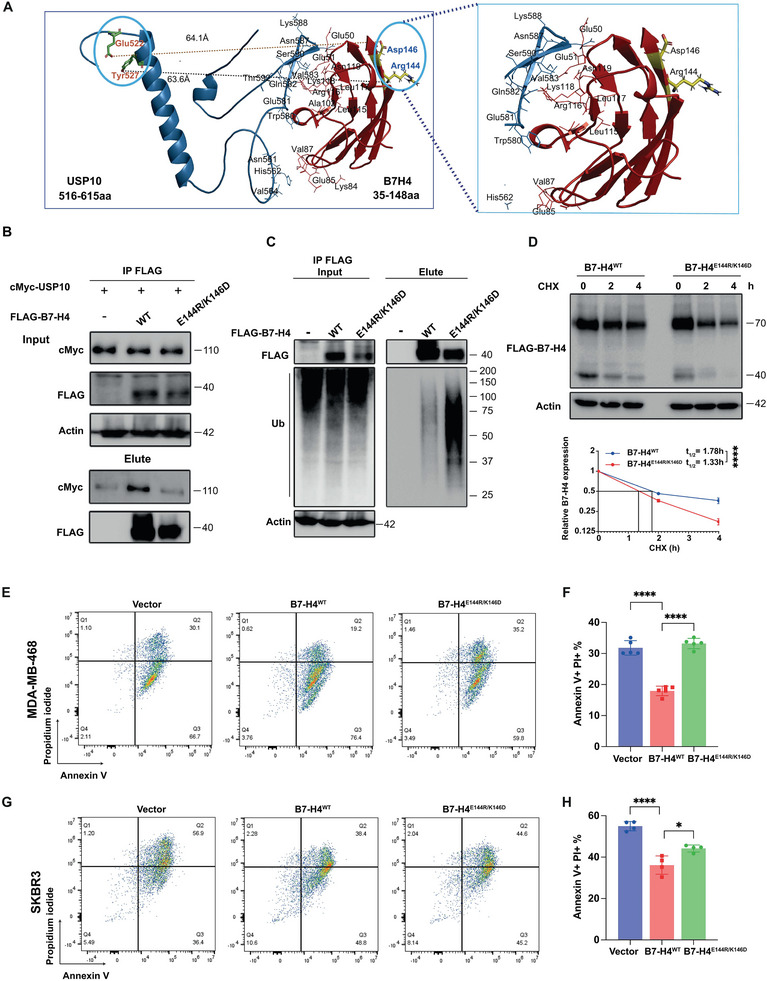
Structural modeling identified the critical role of E144/K146 on B7‐H4 in mediating USP10‐mediated stabilization of B7‐H4. A) The structure model of USP10‐B7‐H4^E144R/K146D^. Mutation of B7‐H4 (red) residue Glu144 and Lys146 to Arg144 and Asp146 highlighted as sticks and colored in yellow. The double mutation disrupts the interaction: Glu144 (yellow)‐Tyr527(green) interaction, and Lys146(yellow)‐Glu522(green) in the previous interface with USP10 (sky blue) and shows long distance (63.6 and 64.1 Å) to each other. USP10 binds to a region other than the critical residues of B7‐H4 (left). Zoom in view of the mutations in the B7‐H4 and its hydrophobic interactions with USP10. B,C) Validation of interaction between USP10 with B7‐H4 and B7‐H4^E144R/K146D^ by coimmunoprecipitation of ectopic Flag‐tagged B7‐H4^WT^ and Flag‐tagged B7‐H4^E144R/K146D^ in HEK‐293T cells. The ubiquitylation status of B7‐H4^WT^ and B7‐H4^E144R/K146D^ were also determined using an antibody against ubiquitin. D) MDA‐MB‐468‐B7‐H4^WT^ and MDA‐MB‐468‐B7‐H4^E144R/K146D^ cells were treated with 100 µg mL^−1^ CHX. Cell lysates were collected at indicated time points and followed by measuring B7‐H4 protein turnover. Quantification of B7‐H4 turnover were illustrated in the bottom panel. E–H) Annexin V/Propidium Iodide apoptosis assay shows that in comparison with WT, E121R&K123D mutation leads to increased cancer cell apoptosis in the coculture with human PBMC. MDA‐MB‐468 (E.F) and SKBR3 (G,H) cells expressing either B7‐H4^WT^ and B7‐H4^E144R/K146D^ mutant were cocultured with activated human PBMCs. Representative images of flow cytometry (E, G). Quantitative data show the percentage of cells in late apoptosis (Annexin V+ and PI+) stage (F, H). *p < 0.05 and ****p < 0.0001. Data (mean ± SEM) are representative of at least three independent experiments.

To determine the critical amino acid residues essential for the interaction between USP10 and B7‐H4, we utilized in silico mutation scans to assess the impact of specific mutations on this protein interaction. Our findings indicate that mutations of B7‐H4 residues Glu144 and Lys146 to Arg144 and Asp146, respectively, which yielded the lowest binding energy of −793.8 kJ mol^−1^, significantly disrupted the interaction interface with USP10, highlighting their crucial role in facilitating their binding (**Figure** **5**A). To experimentally validate the direct involvement of the Glu144 (E144) and Lys146 (K146) residues of B7‐H4 in mediating its association with USP10, we engineered mutant FLAG‐B7‐H4^E144R/K146D^ constructs and co‐transfected them with c‐Myc‐USP10 into HEK‐293T cells. Following co‐immunoprecipitation assays, the results illustrated in Figure 5B suggest a substantial reduction of the interaction between the mutant B7‐H4 and USP10. This alteration was also associated with increased ubiquitin linkage (Figure 5C) and accelerated degradation of the mutated B7‐H4 protein (Figure 5D). Moreover, in the context of a cancer cell and PBMC coculture system, MDA‐MB‐468, SKBR3, and HEK‐293T cells expressing the B7‐H4^E144R/K146D^ mutant demonstrated a differential apoptotic response. Compared to cells with vector control, the mutant cells showed reduced apoptosis, while compared to cells expressing B7‐H4^WT^, they exhibited increased apoptosis (Figure 5E–H and Figure S5A,B, Supporting Information). The above observation suggests that the B7‐H4^E144R/K146D^ mutant, despite retaining some immune suppressive function, is compromised in its efficacy due to a deficiency in deubiquitylation. These results collectively underscore the critical role of specific amino acid residues in the functional interaction between B7‐H4 and USP10, with implications for the stability and immune regulatory function of B7‐H4.

### 2.4 Unexpected Elevation of USP10/B7‐H4 Impedes the Effectiveness of Sacituzumab Govitecan through its Immunosuppressive Role in Immune‐Cold Breast Tumors

**Figure 6:**
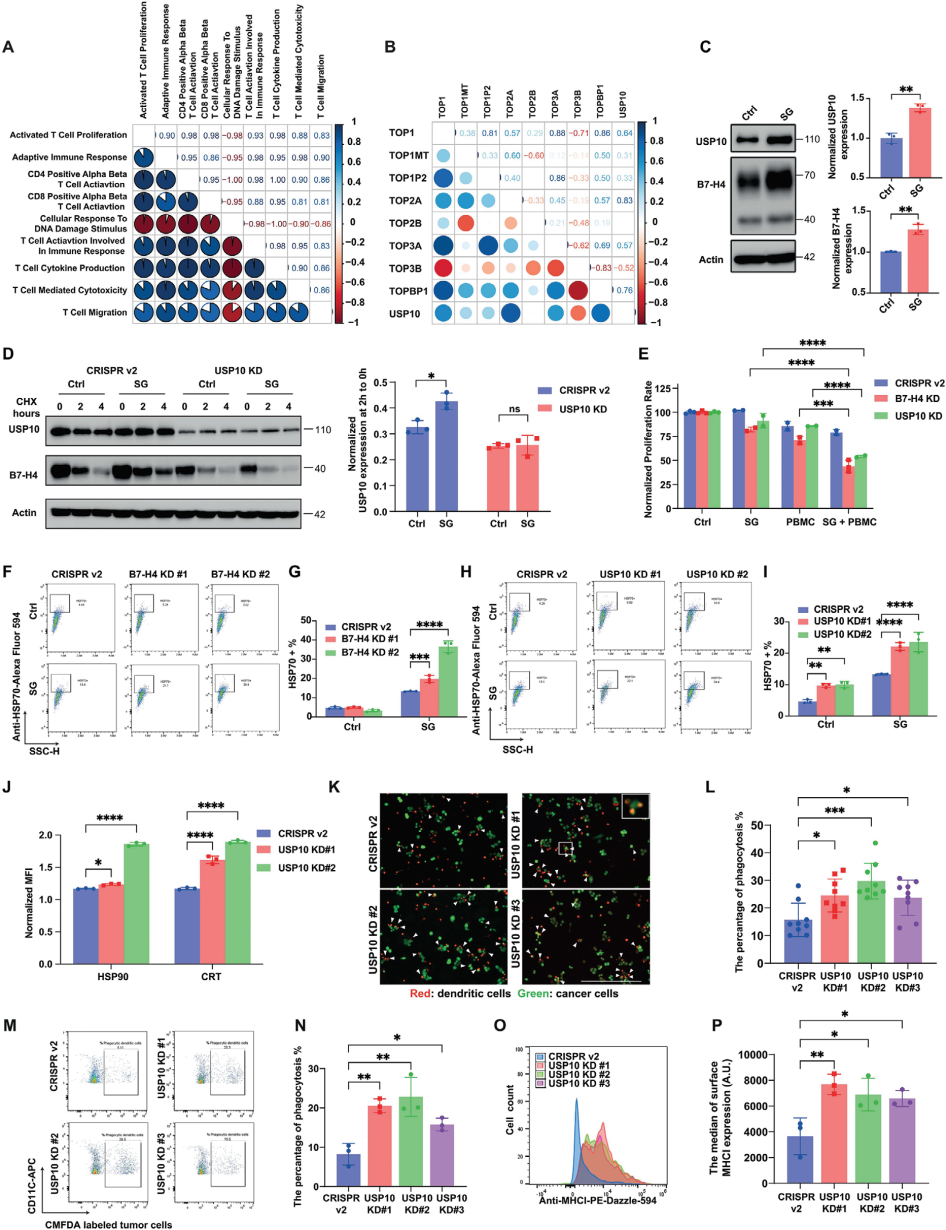
Stabilization of B7‐H4 by USP10 impedes the effectiveness of Sacituzumab govitecan through its immunosuppressive role in immune‐cold breast tumors. A) The MSigDB‐based pathway correlation analysis using GSE72362 database shows that tumor cell related adaptive immune response pathways are negatively correlated with SG payload induced DNA damage response. B) Spearman's rank gene correlation analysis using GSE72362 database shows that USP10 expression is highly positively correlated with DNA topoisomerase expression. C) MDA‐MB‐468 cells were treated with 0.5 µg mL^−1^ SG for 24 h. USP10 and B7‐H4 protein levels were determined by immunoblotting (left panel). Quantification of USP10 and B7‐H4 protein normalized with β‐actin amount in biological triplicates (right panel). D) MDA‐MB‐468 cells either with or without USP10 knockdown were treated with either PBS (control) or 100 ng mL^−1^ SG for 24 h, followed by treatment with 100 µg mL^−1^ cycloheximide (CHX) at specified time points (hours). The left panel shows representative immunoblotting images for B7‐H4. The right panel provides the quantification of B7‐H4 (40 kDa) intensity at 2 h normalized to the level at time 0. E) MDA‐MB‐468‐B7‐H4 KD and MDA‐MB‐468‐USP10 KD cells were cocultured with human PBMCs at Effector (E) to Target (T) ratio (3:1) and treated with SG (0.1 µg mL^−1^), cell proliferation rate was measured by the cell confluence normalized to the corresponding vehicle control of each cell line (%). F–J) MDA‐MB‐468‐B7‐H4 KD and MDA‐MB‐468‐USP10 KD cells were treated with SG (0.5 µg mL^−1^), surface expression of damage‐associated molecular patterns (DAMPs) markers: HSP70, HSP90 and Calreticulin (CRT) was measured by flow cytometry. (F, H) representative flow cytometry images. G, I, J) Quantification of the percentage of positive cells. K,L) The control CRISPR v2 and USP10 knockdown (KD) MDA‐MB‐468 cells were pretreated with SG (100 ng mL^−1^) for 24 h and then cocultured with purified human dendritic cells at a 1:1 ratio. The cancer cells and purified human dendritic cells were stained with cell tracker green CMFDA and red CMTPX, respectively, and subjected to fluorescence imaging after a 3‐h coculture. K) Representative microscopic images. Scale bar = 500 µm. L) Quantification of phagocytosis based on fluorescence microscopic images. The phagocytosis percentage is the number of dendritic cells with engulfed cancer cells, indicated by the colocalization of green and red signals, over the total number of dendritic cells per view. M,N) SG (100 ng mL^−1^) pretreated control CRISPR v2 and USP10 knockdown (KD) MDA‐MB‐468 cells were stained with cell tracker green CMFDA, seeded on a plate, and cultured with human dendritic cells for 2 h. The dendritic cells were then collected and subjected to immunostaining with anti‐CD11c‐APC and analyzed by flow cytometry. M) Representative flow cytometry images. N) Quantification of the percentage of phagocytosis, indicated as the percentage of CMFDA+ dendritic cells. O,P) SG (100 ng mL^−1^) pretreated control CRISPR v2 and USP10 knockdown (KD) MDA‐MB‐468 cells were cultured with the human dendritic cells for 2 h. Dendritic cells were collected and subjected to immunostaining with anti‐MHC‐I‐PE‐Dazzle‐594 and analyzed by flow cytometry. O) Representative flow cytometry image showing the distribution of surface MHC‐I expression levels in dendritic cells. P) Quantification of the median membrane staining of MHC‐I in dendritic cells cocultured with the corresponding cancer cells. *p < 0.05, **p < 0.01, and ***p < 0.001, ****p < 0.0001 by the one‐way or two‐way ANOVA test. Data (mean ± SEM) are representative of at least three independent experiments.

Tumor‐intrinsic genomic instability can be further enhanced by TOP1 inhibitors, such as SN‐38, the payload of SG, to instigate immune responses via T cell activation. Our previous study has shown that increased B7‐H4 expression block chemo agents induced immunogenic cell death.^[^ ^3^ ^]^ In addition, pathway analysis from MsigDB suggests a significant association between USP10 expression and the DNA damage response,^[^ ^25^, ^26^ ^]^ as depicted in Figure S6A, Supporting Information. This observation raises the question of whether DNA damage stimulus, like the payload of SG, impacts the deubiquitylation of B7‐H4 orchestrated by USP10. Pathway correlation analysis (**Figure** **6**A) has revealed that robust DNA repair activity is often accompanied by a negative adaptive immune response and reduced T cell cytotoxicity. Moreover, gene correlation analysis has underscored a strong association between DNA topoisomerases and USP10 levels (Figure 6B and Figure S6B,C, Supporting Information), reinforcing the possible interaction between DNA damage repair mechanisms and immune evasion strategies. In our experimental setup, MDA‐MB‐468 breast cancer cells exposed to SG in addition to other DNA damaging agents showed increased levels of both USP10 and B7‐H4 (Figure 6C and Figure S6D, Supporting Information), suggesting that SG and other DNA‐damaging agents, including those used in systemic chemotherapy, may contribute to an immune‐suppressive milieu governed by B7‐H4. Further investigation into the effects of SG on USP10‐mediated deubiquitylation showed that SG treatment significantly reduced B7‐H4 ubiquitylation (Figure S6E, Supporting Information) and stabilized B7‐H4 protein expression (Figure 6D). To further elucidate the role of USP10 in mediating SG‐induced B7‐H4 upregulation, we treated MDA‐MB‐468 cells with a stable knockdown of USP10 with 100 ng mL^−1^ SG. As shown in Figure S6F–H, Supporting Information and Figure 6D, depletion of USP10 led to unchanged B7‐H4 levels and protein turnover post‐SG treatment, highlighting the critical role of SG in maintaining B7‐H4 stability through USP10. To investigate the mechanism through which SG treatment modulates USP10 expression and subsequently impacts B7‐H4 levels, we assessed both USP10 mRNA levels and protein turnover rate. As shown in Figure S6I,J, Supporting Information, while the SG treatment did not alter the USP10 turnover rate, we observed a significant increase in mRNA levels, suggesting that SG induces USP10 upregulation predominantly through transcriptional activation.

To assess the potential of targeting the USP10/B7‐H4 axis to sensitize breast cancer cells to SG, we conducted coculture assays with MDA‐MB‐468 and HCC70 where B7‐H4 or USP10 was knocked down, followed by SG treatment. The results, particularly illustrated in Figure 6E and Figure S6K–N, Supporting Information, demonstrate that disruption of this axis significantly enhances the anti‐proliferative effects of SG by promoting apoptosis in cancer cells, even at a low effector‐to‐target (E: T) ratio of 3:1. Conversely, restoring B7‐H4 expression in USP10 knockdown (KD) cells significantly mitigated the cytotoxic effects of SG, particularly in the presence of PBMC coculture (Figure S7A–E, Supporting Information). This confirms the crucial role of the USP10‐B7‐H4 axis in modulating the tumor immune response to SG treatment. Furthermore, the increased lethality of B7‐H4 and USP10 depletion tumor cells upon SG treatment appears to be linked to the induction of endoplasmic reticulum (ER) stress and the upregulation of DAMPs, such as calreticulin (CALR), heat shock proteins 70 and 90 (HSP70 and HSP90), as evidenced by flow cytometry analysis (Figure 6F–J). To further characterize how downregulation of the USP10/B7‐H4 axis‐induced DAMPs potentiates immunogenic cell death via enhanced T cell activation, we investigated the role of the USP10/B7‐H4 axis in dendritic cell phagocytosis and subsequent antigen presentation. Purified human dendritic cells were cocultured with either control or USP10 knockdown MDA‐MB‐468 cancer cells pretreated with 100 ng mL^−1^ SG. Real‐time live cell imaging revealed an increased percentage of dendritic cell phagocytosis of cancer cells in the coculture with USP10 KD cells (Figure 6K,L). Consistently, quantitative analysis of dendritic cell phagocytosis via flow cytometry confirmed a similar increase in phagocytosis of USP10 KD MDA‐MB‐468 cancer cells after SG treatment (Figure 6M,N). To assess whether this enhanced phagocytosis was accompanied by improved antigen presentation, potentially leading to T cell priming and activation, we evaluated the membrane expression of MHC‐I on dendritic cells. A significant elevation in MHC‐I membrane expression was detected after coculturing with USP10 KD MDA‐MB‐468 cancer cells compared to controls (Figure 6O,P). These observations suggest that elevated expression of B7‐H4 and USP10 may be a barrier to the effectiveness of SG treatment. Restoring B7‐H4 turnover by inhibiting USP10 could potentially convert a cold tumor immune environment to a more responsive one, thereby increasing SG effectiveness.

### 2.5 Suppression of USP10/B7‐H4 Proteolytic Axis Reinvigorates Therapeutic Efficacy of ADCs through Tuning Immune‐Cold Breast Tumor Hot

**Figure 7:**
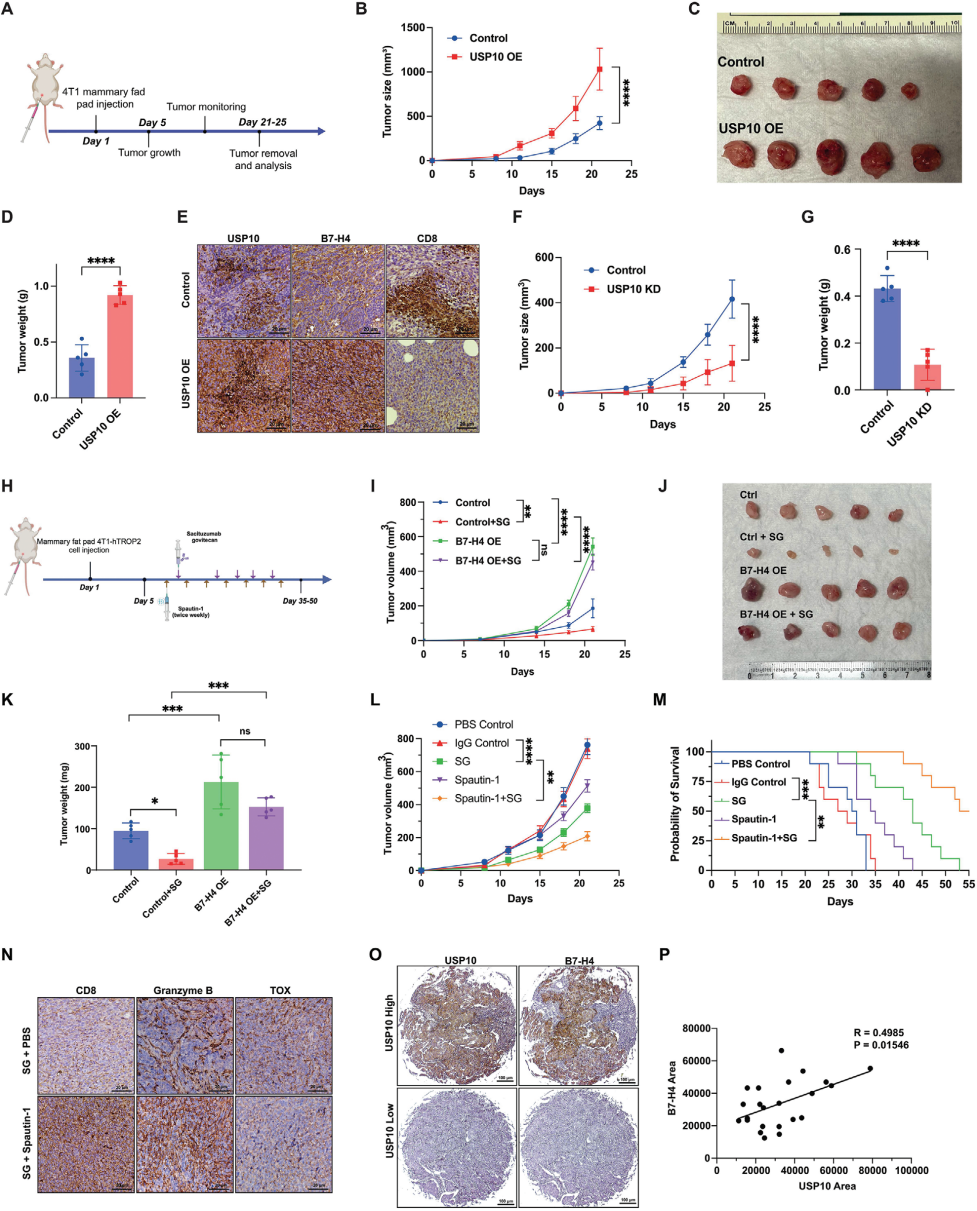
Suppression of USP10/B7‐H4 proteolytic axis in vivo reinvigorates therapeutic efficacy of ADCs through regulating tumor immune response. A–D) 4T1 control and 4T1‐USP10 overexpression breast cancer cells were orthotopically injected into the right fourth mammary gland of the BALB/c wild‐type (WT) mice (A). Phosphate‐buffered saline (PBS) was used in the control group. Tumor growth curve was plotted (B) and tumor was harvested at 21 days after tumor challenge, weight was imaged and measured (C,D). E) Representative images of IHC staining of mouse USP10, mouse B7‐H4 and mouse CD8+ cells level of control and USP10 OE mouse tumors. F,G) 4T1 control and 4T1‐USP10 KD tumors were harvested 21 days after tumor challenge and analyzed. Tumor growth curve was plotted (F) and tumor weight (G) was measured at the end point. H) Schematic diagram of 4T1‐hTROP2, where the basal mouse TROP2 was replaced with a human counterpart, and 4T1‐hTROP2 cells were orthotopically injected into the left fourth mammary fat pad and allow to grow ≈100 mm^3^, followed by injection of spautin‐1 (20 mg kg^−1^, i.p.) for two times per week, and SG (10 mg kg^−1^, i.p.) for three times. PBS and IgG control were used in control groups. I–K) 4T1‐hTROP2 and 4T1‐hTROP2 mB7‐H4 OE breast cancer cells were orthotopically injected into the left fourth mammary fat pad followed by injection of SG (10 mg kg^−1^, i.p.) for three times. Tumor growth curve was plotted (I) and tumor was harvested after tumor challenge, weight was imaged (J) and measured (K). L,M) 4T1‐hTROP2 cells were orthotopically injected into the left fourth mammary fat pad and allow to grow ≈100 mm^3^, followed by injection of spautin‐1 (20 mg kg^−1^, i.p.) for two times per week, and SG (10 mg kg^−1^, i.p.) for three times. PBS and IgG control were used in control groups. The tumor growth was monitored twice per week. Tumor growth (L) and survival curve (M) of the mice were plotted. N) Representative images of IHC staining of mouse CD8, granzyme B and TOX level of 4T1‐hTrop2 mouse tumors after the treatment of SG treatment or SG + Spautin‐1 combination. Scale bar = 20 µm. O,P). USP10 expression level and B7‐H4 expression level were analyzed and quantified by IHC using TMA consisting of TNBC samples. Representative IHC images (K). Quantification of USP10 and B7‐H4 expression (L). *p < 0.05, **p < 0.01, ***p < 0.001, and ****p < 0.0001 by the two‐way ANOVA test. Data (means ± SEM) are representative of at least two independent experiments with five to ten independently analyzed mice per group.

To investigate the influence of USP10 on tumor progression through the regulation of B7‐H4 in vivo, we engineered murine 4T1 breast cancer cell lines to alter USP10 expression. We established USP10 overexpression (USP10‐OE) and knockdown (USP10‐KD) in 4T1 cells (Figure S8A,B, Supporting Information), using cells with an empty vector as the control group. The control 4T1 cells and 4T1 cells overexpressing USP10 were then injected subcutaneously into the mammary fat pads of female BALB/c mice. Tumor volumes were periodically measured with calipers and calculated with the formula V = (W^2 × L)/2, where V represents the tumor volume, W is the width, and L is the length of the tumor. After 21 days post‐injection, mice injected with USP10‐OE cells exhibited a substantial increase in tumor growth rate and final tumor weight (**Figure** **7**A–D). Additionally, immunohistochemical staining of tumor tissues demonstrated that upregulation of the USP10‐B7‐H4 axis dampened the tumor immune response, evidenced by a decreased CD8+ T cell population (Figure 7E). In contrast, mice injected with USP10‐KD cells displayed significantly inhibited tumor growth and reduced tumor weight compared to the control group (Figure 7F,G). To determine whether alteration of USP10 could directly affect tumor cell proliferation independently of immune system interactions, we conducted experiments using nude mice bearing 4T1‐USP10‐OE or 4T1‐USP10‐KD xenografts. Our results demonstrated that neither USP10 overexpression nor USP10 knockdown exhibited significant differences in tumor growth in the 4T1 xenograft mouse model (Figure S8C–H, Supporting Information).

To evaluate the impact of elevated B7‐H4 on the efficacy of SG, we overexpressed B7‐H4 in 4T1 cells engineered to express human TROP2 (4T1‐hTROP2) (Figure S8I, Supporting Information and Figure 7H–K). Results shown in Figure 7H–K indicate that 4T1 cells with increased B7‐H4 levels exhibited significantly reduced sensitivity to SG, leading to diminished tumor growth inhibition compared to the parental 4T1‐hTROP2 cells. Similarly, 4T1‐hTROP2 cells with USP10 knockdown and subsequent reintroduction of B7‐H4 also showed notable increases in tumor growth and enhanced resistance to SG (Figure S8J–M, Supporting Information), confirming the axis's critical role in TNBC progression and response to SG therapy. Furthermore, to explore the therapeutic potential of targeting the USP10/B7‐H4 axis in TNBC, we investigated the anti‐tumor effects of the USP10 inhibitor spautin‐1, both alone and in combination with the Trop‐2 directed antibody‐drug conjugate, SG, in a TNBC 4T1 model engineered to express human TROP2. The findings, presented in Figure 7L,M, demonstrate that the combination of spautin‐1 with SG significantly inhibited tumor growth and extended the median survival from 43 to 55 days compared to each agent alone. Additionally, at the end of the 60‐day experimental monitoring phase, there were still 5 mice alive in the combination group. To evaluate the impact of inhibiting the USP10‐B7H4 axis on T cell function, we analyzed tissue samples from 4T1‐hTROP2 TNBC tumors. The findings, presented in Figure 7N, showed that the combination of SG and spautin‐1 markedly increased CD8+ T cell infiltration and activation. Additionally, we observed a moderate decrease in T cell exhaustion, as evidenced by reduced Tox staining, when compared to treatment with SG alone. These outcomes suggest the therapeutic potential of targeting the USP10‐B7‐H4 axis pharmacologically to enhance immunogenic cell death in response to the TOP1 inhibitor, the payload of SG, particularly in TNBC subtypes characterized by high B7‐H4 expression and low immunogenicity.

To determine the clinical relevance of B7‐H4 proteolytic regulation on immune response and prognosis in TNBC, we conducted a differential gene expression analysis comparing tumor tissues with adjacent normal tissues. This analysis revealed a significant upregulation of USP10 in breast cancer cases (Figure S9A, Supporting Information). We stratified TNBC patients into TROP2‐high and TROP2‐low groups and observed a positive correlation between high TROP2 expression and increased USP10 expression (Figure S9B, Supporting Information). This suggests that USP10 may play a pivotal role in mediating the increase in B7‐H4 expression in the context of high TROP2 levels and SG treatment. Additionally, immunohistochemistry (IHC) analysis on a tissue microarray comprising 110 distinct breast tissue specimens further confirmed a positive correlation between USP10 and B7‐H4 expression levels (Figure 7O,P). Furthermore, immune profiling using CIBERSORT, as shown in Figure S9C–E, Supporting Information, indicated that tumors with increased USP10 expression exhibited a reduced CD8+ T cell population. This was associated with lower overall lymphocyte infiltration and heightened tumor proliferation rates. Moreover, an analysis of TNBC patient data from the TCGA database demonstrated that high USP10 expression was associated with worse survival outcomes, even in cases with a high presence of cytotoxic T lymphocytes (Figure S9F–I, Supporting Information).

**Figure 8:**
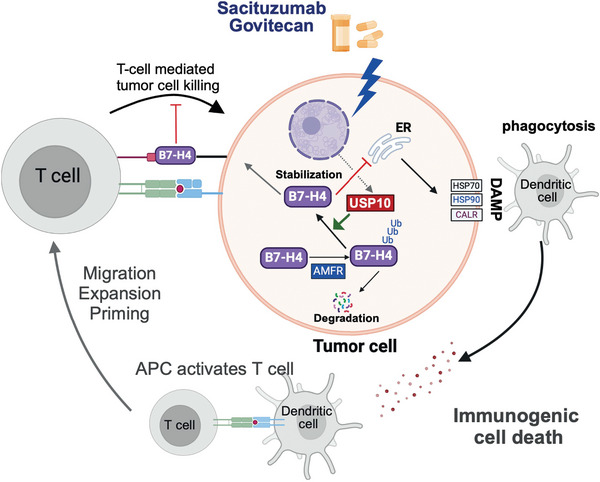
Proposed working model. In immune‐cold triple‐negative breast cancers, elevated B7‐H4 expression is a barrier that attenuates tumor immune responses and impedes the efficacy of targeted therapies such as sacituzumab govitecan. B7‐H4 levels is governed by a “yin” and “yang” proteolytic regulation through E3 ligase AMFR and deubiquitinase USP10, creating a delicate balance between degradation and stabilization. The stabilization of B7‐H4 by USP10 leads to diminished immune activity and lessened response to ADCs. Pharmacological inhibition of USP10 restore B7‐H4 turnover, reviving tumor immunogenicity and enhancing the therapeutic impact of Sacituzumab govitecan.

Overall, our results demonstrate that USP10 plays a critical role in regulating B7‐H4, which, in turn orchestrates tumor immune evasion by stabilizing protein expression through deubiquitylation. Suppression of USP10/B7‐H4 proteolytic axis could be a novel strategy to reduce B7‐H4 and improve immune response in breast cancer treatment (**Figure** **8**).

## 3 Discussion

Based on the integration of unbiased big data analyses, mass spectrometry and experimental validation including preclinical models, we identified the impact of the USP10/B7‐H4 proteolytic axis in orchestrating tumor immunogenicity and the therapeutic efficacy of ADCs. We demonstrated the cellular protein levels of B7‐H4 are dictated by the proteolytic machineries USP10 and AMFR, whose deregulation dampens tumor immune activity and cytotoxic effectiveness of ADCs. Pharmacological inhibition of B7‐H4 deubiquitylation restored B7‐H4 degradation, thereby transforming immune‐cold TNBC tumors into immune‐hot tumors. This further promoted the onset of ADC‐induced immunogenic cell death and revived the tumor immune response (Figure 8). This study provides a new paradigm to develop strategies to treat immune‐cold TNBC patients.

### 3.1 “Immune‐Cold” Status Impedes Tumor Immunogenicity and Cytotoxic Drug Response

The dedicated interplay between tumor immunology and the therapeutic efficacy of cytotoxic drugs is a pivotal aspect of contemporary breast cancer research. This dynamic is critically influenced by the presence of an “immune cold” tumor microenvironment, characterized by diminished immunogenicity and a weakened cytotoxic response to therapeutic agents.^[^ ^27^, ^28^ ^]^ This environment severely hampers the effectiveness of cytotoxic and chemotherapeutic strategies, as the lack of immune activity results in deficient inflammatory signaling and antigen presentation, which are essential for the recognition and destruction of tumor cells by effector immune cells.^[^ ^29^ ^]^ As a result, tumors fortify themselves against the intended effects of cytotoxic drugs. This immune evasion leads to a resilience that enables the tumor to withstand cytotoxic therapeutic interventions, underscoring the urgency to unravel the underlying mechanisms that govern this evasion.

The “cold” nature of a tumor microenvironment is not a passive state but rather an active orchestration by the tumor to evade immune detection, which effectively protects the tumor by shielding it from the immune system's surveillance.^[^ ^30^, ^31^ ^]^ ADCs provide an approach to overcome the challenge and re‐educate the immune system to detect the tumor again by engineering and delivering potent cytotoxic agents directly to cancer cells with remarkable specificity.^[^ ^13^, ^32^ ^]^ ADCs offer a clear advantage over traditional chemotherapeutics by utilizing a targeted mechanism that limits systemic toxicity. The multifaceted action of ADCs goes beyond simply inducing apoptosis in cancer cells through their toxic payloads. They also impede receptor‐mediated signaling pathways via the attached antibody and induce a “bystander effect,” attracting immune effector cells to the tumor. This process reignites antitumor immunity and transforms a “cold” microenvironment into a “hot” one, which is beneficial for overcoming therapeutic resistance in TNBC.^[^ ^11^, ^33^ ^]^ ADCs induced immunogenic cell death can promote antigen uptake and migration of tumor‐resident dendritic cells to the tumor‐draining lymph nodes, enhancing T cell priming and expansion.^[^ ^34^, ^35^ ^]^

However, the function of these immune cells can be hindered by the engagement of immune checkpoint molecules such as PD‐L1/PD1 and B7‐H4.^[^ ^3^ ^]^ While the immune checkpoint inhibitors (ICIs) can unleash and sustain the T‐cell response against the tumor, limited fraction of TNBC patients with PD‐L1 expression and inherent resistance is a barrier affecting its therapeutic effectiveness.^[^ ^36^ ^]^ Therefore, identifying the impact of B7‐H4 and its proteolytic regulatory mechanisms in TNBC offers a potential targeting strategy to sensitize immune‐cold TNBC tumors to ICIs and ADCs.

### 3.2 Unexpected Elevation of B7‐H4 Protein Level Due to its Impaired Proteolysis Attenuates Tumor Immune Response and Susceptibility to Anti‐Cancer Drugs

Our multiomic analyses pinpointed the role of B7‐H4 in orchestrating the immune “cold” status of tumors in a subset of TNBC patients. IHC analyses with various breast cancer cohorts further unveiled the unexpected accumulation of B7‐H4 protein in TNBC patient samples, indicating a correlation with dysregulated B7‐H4 turnover regulation and poor prognosis. Overexpression of the immunosuppressive protein B7‐H4 inhibits T‐cell activation and tumor adaptive immune response.^[^ ^14^, ^15^, ^16^, ^17^ ^]^ Intriguingly, our studies also revealed the concurrent upregulation of TROP2, the target receptor for the antibody‐drug conjugate sacituzumab govitecan, in patients with elevated levels of B7‐H4, suggesting the potential connection between B7‐H4 with the therapeutic effectiveness of SG. Our recent studies also explored the impotence of B7‐H4 in regulating immunogenic cell death, where B7‐H4 interferes with the PERK‐mediated phosphorylation of eIF2α, a step essential for the surface exposure of CALR, acting as a “danger” or “eat‐me” signal for phagocytic cells.^[^ ^3^ ^]^

Consequently, B7‐H4 undermines the cascade of events that would typically lead to the release of DAMPs associated with immunogenic cell death (ICD), preventing the phagocytosis of tumor cells by dendritic cells and ultimately obstructing T cell recruitment to the tumor site.^[^ ^3^ ^]^ In this regard, the elevated B7‐H4 expression may pose a significant barrier for a subgroup of TNBC patients receiving SG treatment which was also validated in this study, where TNBC cells with high B7‐H4 exhibited significantly lower response to SG treatment. Given this context, the upregulation of B7‐H4 could represent a significant obstacle for a subset of TNBC patients undergoing SG therapy. Therefore, the destruction of B7‐H4 could boost tumor immunogenicity and promote ICD‐induced by SG, especially for the subset of B7‐H4^high^ TNBC patients.

### 3.3 Stabilization of B7‐H4 by USP10 Limits the Effectiveness of Sacituzumab Govitecan in Breast Cancer Treatment

We demonstrated the unexpected accumulation of B7‐H4 in TNBCs is attributed to its dysregulated turnover regulation. To maintain homeostasis, B7‐H4 needs to be precisely regulated by posttranslational modifications. While degradation of B7‐H4 is governed by ubiquitin E3 ligase AMFR, stabilization can be achieved at two levels, including B7‐H4 glycosylation and deubiquitylation. Previously, we showed that glycosylation of B7‐H4 alters its protein conformation, which changes the recognition and interaction of the E3 ligase with B7‐H4.^[^ ^3^ ^]^ In the present study, the mass spectrometry identified the USP10 as a deubiquitinase that upregulates B7‐H4 through counteracts the E3 ligase‐mediated ubiquitylation and degradation, resulting in inhibition of tumor immune response and cellular response to ADC treatment. Stabilization of B7‐H4 by USP10 enhances the “cold” tumor environment and potentially reduces the therapeutic efficacy of SG. Moreover, our data suggest that treatment with SG may promote a feed‐forward loop that upregulates USP10 expression, thereby leading to further elevation of B7‐H4 level, and potentially reinforcing the immune evasion facilitated by this protein. Our work established the regulatory circuity for “ying” and “yang” regulation of B7‐H4 and revealed that deregulated USP10 in the context of etiological status is a key for the accumulation of B7‐H4 in TNBCs observed by IHC and cohort studies. Pharmacological inhibition of the USP10/B7‐H4 axis could be an effective strategy to convert “cold” TNBC tumors into more immunogenic ones and augment the effectiveness of SG in treating TNBC patients.

## 4 Experimental Section

### Bioinformatics Analysis

Public cancer patient datasets were analyzed with established bioinformatics methods.^[^ ^37^ ^]^ This work analyzed several public datasets from TCGA and CPTAC related to breast cancer using established bioinformatics methods^[^ ^38^ ^]^ and grouped into different subtypes according to PAM50 signature. The genes were clustered according to the difference between the average expression of each subtype and the average expression of other subtypes. Protein expression levels corresponding to the listed immune‐related proteins were analyzed and heatmap were generated. This work evaluated Spearman's correlation coefficients to assess the significance of the correlations between gene expression values in TNBC samples. For the hierarchical clustering of enriched terms, this work employed the average linkage method and visualized the correlation coefficients in a heatmap. Patient survival analysis and expression analysis were conducted in Python based on the TCGA PacCancer Atlas data sets available through the NCI GDC Data Portal. The immune infiltration analysis, immunogenicity analysis and pathway enrichment analysis were done by using TIMER2.0, TIDE or CAMOIP web‐based analysis servers.^[^ ^22^, ^39^, ^40^ ^]^ Molecular Signature Database (MSigDB),^[^ ^41^ ^]^ KEGG pathway,^[^ ^42^ ^]^ GO,^[^ ^43^ ^]^ and Reactome gene sets^[^ ^44^ ^]^ were used for pathway and functional enrichment analyses. All software used in the present study are available in the Python Package Index (PyPI) (https://pypi.org/) and/or other relevant Python repositories.

### Cell Culture and Chemical Reagents

HEK‐293T, MDA‐MB‐468, MDA‐MB‐231, SKBR3, 4T1, HCC1599, HCC70, and HCC1937 were obtained from American Type Culture Collection (ATCC) (Manassas, VA). 4T1‐hTROP2 cells, where the basal mouse TROP2 was knocked out and replaced with human TROP2. HEK‐293T, MDA‐MB‐468, MDA‐MB‐231, SKBR3, HCC70 & 4T1 were cultured in Dulbecco's Modified Eagle Medium (Corning, 10‐017‐cv), and HCC1599 & HCC1937 were cultured in RPMI‐1640 Medium (Corning, 10‐040‐CV), respectively supplemented with 10% fetal bovine serum (FBS) and 1% of streptomycin (100 U mL^−1^) and penicillin (100 U mL^−1^) at a humidity incubator at 37 °C with 5% CO_2_. Other chemicals used in cell culture: Cycloheximide (Cayman, 14 126), Sacituzumab Govitecan (TRODELVY), Spautin‐1 (Selleckchem, S7888), Olaparib (Sigma‐Aldrich, SML3705), Doxorubicin (Sigma‐Aldrich, D1515) and Wu‐5 (AOBIOUS, AOB11528).

### Plasmids Information

The N‐FLAG tag human B7‐H4 (HG10738‐NF) were ordered from Sino Biological Inc. LentiCRISPRv2 puro (Plasmid #98 290) was purchased from Addgene and used for the vector control for CRISPR Knock‐down. For CRISPR knockdown of B7‐H4 and USP10, sgRNA guide sequences were cloned into LentiCRISPRv2 puro following a published PCR free method.^[^ ^45^ ^]^ The sgRNA sequence targeting human B7‐H4: 5′‐TCGGGGAGCCTCACACCGCA‐3′ and 5′‐ATTGGAGACTTCCGAGAAGT‐3′. The sgRNA sequence targeting human USP10: 5′‐CTTACCTCAACTGAAGATCG‐3′ and 5′‐CATGGCCCTCCACAGCCCGC‐3′. The sgRNA sequence targeting USP36: 5′‐CGGGCTACAGCTGCCATGC‐3′; USP17: 5′‐TCAATCGAAATACAAGTGT‐3′; USP42: 5′‐TTCCCGTTAGGACTCGACA‐3′; USP30: 5′‐GCCAAGGGAACGTTGAACG‐3′;USP49: 5′‐GGCGTGGTCCTCAATATAG‐3′; USP44: 5′‐TGTTGGGCGTAACCACGAA‐3′; USP3: 5′‐TCTACTAACAAATCGTAAG‐3′; USP28: 5′‐ATTTCAGCATGTCCGGGAG‐3′; USP25: 5′‐AAGGGGAATCGTTTGGGAC‐3′; and USP13: 5′‐ ATCCACCCTCTCCGTGTAG‐3′ and 5′‐CGCTTATGAACTAACGAGA‐3′; The successful cloning of sgRNA guide sequences was validated by sequencing with the primer 5′‐GAGGGCCTATTTCCCATGATT‐3′. The N‐cMyc tag human USP10 (#184 034) were purchased from Addgene and then corresponding cMyc‐USP10 coding sequence was cloned into lentiviral vector Phage‐PGK‐Flag‐via Gibson assembly (ClonExpress II One Step Cloning Kit C112 from Vazyme) and validated by sequencing. For USP10 and B7‐H4 fragments, the indicated coding sequence was amplified by PCR, cloned into pIRES and pCI vector, respectively and validated by restriction enzyme digestion and sequencing. For B7‐H4 mutagenesis, the corresponding mutations were introduced with primers by PCR amplification, ligated via Gibson assembly and validated by restriction enzyme digestion and sequencing. For mouse USP10 knockdown, CRISPRV v2 sgRNA for mouse ups10‐1 and ups10‐3 were purchased from Sigma Aldrich. For mouse USP10 overexpression, mouse usp10 coding sequence in pcDNA3 vector was purchased from Sigma Aldrich, cloned into lentiviral vector Phage‐PGK‐HA and validated by sequencing.

### Cell Transfection

Cells were transfected with PEI STAR (TOCRIS) Transfection Reagent following the user manual. Generally, cells were seeded the day before transfection to reach 50–80% confluence on the day of transfection. DNA and PEI STAR were mixed in Opti‐MEM medium and added to the cells after 10 min‐incubation at room temperature. Cells were collected for analysis 2–3 days after transfection.

### Stable Cell Line Construction with Lentivirus Transduction

Lentiviral particles were generated by co‐transfection of the expression plasmid and lentiviral package plasmids (pVSV‐G, pRRE, and pRSV‐REV) into HEK‐293T with PEI STAR (TOCRIS). Culture media were harvested 48 h after transfection, filtered through 0.45‐µm filters, mixed with polybrene and added to the targeting cell lines for transduction. 2–3 days after transduction, the stable cell lines were established by puromycin or hygromycin selection.

### Purification of B7‐H4 Complex and Mass Spectrometry

MDA‐MB‐468 cells with stable expression of FLAG‐B7‐H4 were lysed with NP40 buffer (1% NP‐40, 10% glycerol, 25 mmol L^−1^ Tris‐HCl (pH 7.9) and protease inhibitor cocktails. B7‐H4‐interactome was purified by immunoprecipitating FLAG‐B7‐H4 with anti‐FLAG M2 agarose beads (Sigma, A2220) and washed four times with TBST buffer [137 mmol L^−1^ NaC1, 20 mmol L^−1^ Tris‐HCl (pH 7.6), 0.1% Tween‐20]. The interactome was eluted with 3× FLAG peptide (Sigma, F4799) in TBS buffer. The elute was then separated on SDS‐ PAGE followed by Coomassie blue staining. The elute was sent for mass spectrum analysis.^[^ ^46^ ^]^

### Immunoblotting

Cells were lysed with 1X Laemmli sample buffer (Biorad, 1 610 737), boiled at 95 °C for 5 min, and centrifuged at 14 000 × g for 15 min at 4 °C to remove the undissolved cell debris. Protein concentration of cell lysate was measured with the BCA assay kit (Thermo Fisher Scientific, 23 225). 20 µg protein was resolved on SDS‐PAGE and transferred to nitrocellulose membrane. Membranes were blocked with PBST containing 5% skim milk for 2 h, incubated with various primary antibodies at 4 °C overnight followed with 5 min‐washing with PBST for four times and the corresponding horseradish peroxidase (HRP) conjugated secondary antibody at room temperature for 2 h followed with 5 min‐washing with PBST for four times. Chemiluminescence substrate was applied using Clarity Western ECL Substrate (Biorad, 1 705 061). Blots were imaged using the ChemiDoc Touch Imaging System (Bio‐Rad). Semiquantification of Chemiluminescence was performed using ImageLab (Bio‐Rad). Primary antibodies used in immunoblotting: hB7‐H4 (Cell Signaling Technology, 14 572), mB7‐H4 (Abcam, ab269455), USP10 (Cell Signaling Technology, 8501), USP13 (Cell Signaling Technology, 12 577), TROP2 (ABclonal, 20 824), FLAG (Cell Signaling Technology, 14 793), HA (Cell Signaling Technology, 3724), Ubiquitin (Cell Signaling Technology, 43 124), cMyc (Santa Cruz, sc789). Secondary antibodies used in immunoblotting: Anti‐Rabbit‐HRP (Promega, w4018) and Anti‐Mouse‐HRP (Promega, w4028).

### Immunoprecipitation

Cells were lysed at 4 °C in ice‐cold cell lysis buffer (25 mM Tris‐HCl, 150 mM NaCl, 1 mM EDTA, 1% NP40, 5% glycerol) with protease inhibitor cocktail (SIGMAFAST, S8830) for 1 h. Cell lysates were cleared by centrifugation at 13 000 × g for 30 min. Protein concentration in the supernatant was determined using the BCA Protein assay Kit (Pierce, 23 225). For immunoprecipitation, cell lysate was incubated with anti‐FLAG M2 agarose affinity gel (Sigma, A2220) or anti‐cMyc agarose affinity gel (Sigma, A7470) overnight at 4 °C on a rotator and washed with PBS for five times. The complex was eluted with 100 µg mL^−1^ 3X Flag peptides in PBS for 30 mins or boiling in 1X Laemmli sample buffer for 5 min.

### Structure Modeling and Docking Analysis

Structure modeling of full length human B7‐H4 and USP10 sequence was performed through the SWISS‐MODEL and AlphaFold approach using (alphaFold2.ipynb) web‐based server and structural coordinates of AMFR downloaded from PDB (PDB ID: 2LXH).^[^ ^47^ ^]^ All cocrystallized hetero atoms, including water molecules and cocrystallized ligand, were pulled out from the original coordinates. Further, the structure‐based molecular docking and their binding pattern was explored by using the molecular docking approach. There are full length variants of B7‐H4 and USP10, but the region where the interaction of B7‐H4 and USP10 occurs was selected and used for molecular docking simulations. Structural coordinates of B7‐H4 fragments covering the Ig‐like V‐type domain His‐35 to Gly‐148, and USP10 domain Ser‐516 to Tyr‐615, respectively. The docking was performed using the ClusPro 2.0 (https://cluspro.bu.edu/login.php) and HADDOCK 2.4 webserver,^[^ ^48^, ^49^ ^]^ a docking program based on the Fast Fourier Transform (FFT) correlation approach.^[^ ^50^ ^]^ The docking output was filtered based on the interacting residue and energy values, and then top docked conformations (Cluster 3 docking poses) selected and further mutate the Lys‐146, Glu‐144 of B7‐H4 and analyzed the interaction using PyMOL and Discovery Studio Visualizer for their possible interactions.^[^ ^51^ ^]^

### Apoptosis Assay

MDA‐MB‐468 or HEK‐293T cells were seeded 1 day before coculture. Human PBMCs were obtained from donors were thawed and resuspend with 1 ng mL^−1^ anti‐human CD3 (BioLegend, 300 438) and 1 ng mL^−1^ anti‐human CD28.2 (BioLegend, 302 934) for T‐cell activation and added to the seeded cells for at 3:1 ratio. Prepare extra samples for PBMC only control, cancer cells only control as well as single stain control. After 40 h co‐culture, cells were collected for staining with FITC Annexin V Apoptosis detection kit with PI (BioLegend, 640 914) and PE anti‐human‐CD‐45 (BioLegend, 304 039) which was used to stain PBMCs. Cells were analyzed by flow cytometry with proper machine setting and data were analyzed with FlowJo software (BD Biosciences).

### Phagocytosis Assay

Tumor cells were stained with 10 µM CellTracker Green CMFDA Dye (ThermoFisher Scientific, C7025) at 37 °C for 30 min, seeded on the plates, treated with 100 ng mL^−1^ sacituzumab govitecan (SG) for 24 h to induce DAMPs, rinsed with PBS twice to remove the drug and then cocultured with Human monocyte derived dendritic cells (Zen‐Bio, SER‐MODC‐F) at 1:1 ratio for 2–3 h. Dendritic cells were collected, stained with Zombie Yellow, blocked with Human TruStain FcX (BioLegend, 422 302), APC anti‐human CD11c antibody (BioLegend, 980 604) and PE/Dazzle 594 anti‐human HLA‐A2 Antibody (BioLegend, 343 334) and subjected to flow cytometry. For fluorescence imaging, dendritic cells were stained with CellTracker Red CMTPX Dye (ThermoFisher Scientific, C34552) at 37 °C for 30 min before the coculture with cancer cells and imaged with BioTek LionHeart fluorescence microscope.

### T‐Cell Function Analysis with Flow Cytometry

Breast cancer cells were seeded 1 day before coculture. Human PBMCs obtained from donors were thawed and resuspend with 1 ng mL^−1^ anti‐human CD3 (BioLegend, 300 438) and 1 ng mL^−1^ anti‐human CD28.2 (BioLegend, 302 934) for T‐cell activation and added to the seeded cells for at 5:1 ratio. 40 h later, PMA/INOmycin/BFA (Biolegend, 423 303) was added at 1:500 dilution and incubated for 3–4 h before cell collection. Cells were collected and stained with Zombie yellow, then CD45 (Biolegend, 304 039), CD4 (Biolegend, 317 433), and CD8 (Biolegend, 344 722), then permeabilized, fixed and stained antibody for INF‐γ (Biolegend 502 528), TNF‐α (Biolegend, 502 947), and Ki67 (Biolegend, 350 515) followed by washing. Cells were analyzed by flow cytometry CYTEK AURORA with proper machine setting. Zombie yellow negative, CD45+, CD8+ cytotoxic T cells were selected for functional analysis with FlowJo software (BD Biosciences).^[^ ^37^ ^]^

### Protein Measurement by Flow Cytometry

Single cell suspensions were washed with PBS, then fixed with 4% formaldehyde in PBS. Cells were washed with PBS twice and before the incubation with the indicated antibodies with fluorophore conjugation. For the staining of cell surface protein, the staining was performed without membrane permeabilization. For staining of the whole cell, cells were permeabilized with 0.1% Triton X‐100. Cells were washed with PBS and analyzed by flow cytometry CTTEL AURORA with proper machine setting and data were analyzed with FlowJo software (BD Biosciences). Antibodies used for protein measurement by flow cytometry: hB7‐H4 (BD Pharmingen, 562 507), HSP90 (Cell Signaling Technology, 4877) HSP70 (Cell Signaling Technology, 4873), Calreticulin (Cell Signaling Technology, 12 238).

### Immunofluorescence

Cells grown on coverslip were fixed with 4% paraformaldehyde in PBS for 20 min and permeabilized by 0.1% Triton X‐100 for 15 mins at room temperature. The indicated antibodies diluted in PBST with 0.2% BSA with the dilution factor recommended by the manufacture were incubated with cells overnight at 4 °C. After being washed with PBST 10 min × 3, cells were incubated with Alexa 488 anti‐rabbit/594‐anti‐mouse IgG 1:500 diluted in PBST with 0.2% BSA for 1 h. Cells were washed, stained with 1 ng mL^−1^ DAPI in PBS, washed, mounted, and imaged with BioTek LionHeart fluorescence microscope. Primary antibodies used in immunofluorescence: hB7‐H4 (Sigma, HPA054200) and USP10 (Cell Signaling Technology, 8501). Secondary antibodies used in immunofluorescence: anti‐Mouse‐ Alexa Fluor 488 (Fisher Scientific, A11029 and anti‐Rabbit‐ Alexa Fluor Plus 594 (Fisher Scientific, A32740).

### Immunohistochemistry

The human breast cancer tissue array BC081116d were purchased from TMA (US Biomax Inc.), including invasive ductal carcinoma cases (grade 2, n = 28; grade 3, n = 25) and medullary carcinoma cases (n = 9), multiplex IHC staining of USP10, B7‐H4 and CD8 using the Opal multiple color IHC kit (Akoya Biosciences) as described previously.^[^ ^37^ ^]^ Briefly, TMA slides were deparaffinized, re‐hydrolyzed, implemented antigen retrieval in AR9 retrieval buffer (Akoya Biosciences) followed by staining procedures. Briefly, the slides were blocked, incubated with primary antibodies, and incubated with the secondary HRP‐ conjugated antibodies. Heating in AR6 retrieval buffer (Akoya Biosciences) was applied after each staining cycle to release the bounded primary and second antibodies. After the staining procedures, the slide was counter‐ stained with DAPI. Primary antibodies used in immunohistochemistry: hB7‐H4 (Cell Signaling Technology, 14 572), mB7‐H4 (Fisher Scientific, AF2154), USP10 (Cell Signaling Technology, 8501), TOX (Cell Signaling Technology, 73 758), Granzyme B (Cell Signaling Technology, 46 890), mCD8 (Cell Signaling Technology, 98 941) and hCD8 (Cell Signaling Technology, 85 336).

### Duolink Proximity Ligation Assay

The colocalization of B7‐H4 and USP10 was analyzed by the proximity ligation assay by using Duolink In Situ PLA Kit (Sigma‐Aldrich) and following the manufacturer's protocol. In brief, cells were plated on glass coverslips and fixed with 4% formaldehyde and permeabilized with 0.1% Triton X‐100. Cells were then washed with PBS, incubated with blocking buffer followed by overnight primary antibody incubation at 4 °C. Cells were then incubated with PLA probes, ligation mix, amplification mix and washed twice. The coverslips were mounted using the Duolink In Situ Mounting Medium followed by imaging the cells with BioTek LionHeart fluorescence microscope.

### RNA Extraction and qPCR

Total RNA was extracted using TRIzol reagent (Invitrogen) according to the manufacturer's instructions. The complementary DNA synthesis was performed using SuperScript One‐Step RT‐PCR (Invitrogen). Real‐Time Quantitative Reverse Transcription PCR was used to quantify genes by SYBR Green (Bio‐Rad), and relative abundance of each mRNA was normalized to beta‐actin mRNA. The primers for qPCR of B7‐H4: 5′‐TCTGGGCATCCCAAGTTGAC‐3′ and 5′‐TCCGCCTTTTGATCTCCGATT‐3′. The primers for qPCR of USP10: 5′‐ATTGAGTTTGGTGTCGATGAAGT‐3′ and 5′‐GGAGCCATAGCTTGCTTCTTTAG‐3′. The primers for qPCR of ACTB: 5′‐AAGATCATTGCTCCTCCTGAGC‐3′ and 5′‐CATACTCCTGCTTGCTGATCCA‐3′.

### In Vivo Tumor Challenge

BALB/C (8‐week‐old female) were purchased from The Jackson Laboratory. Engineered 4T1 control, 4T1 USP10 KD, 4T1 USP10 OE and 4T1‐hTROP2 breast cancer cells (1 × 10^6^) were injected subcutaneously into the mammary fat pad of female BALB/C mice on day 0. Tumor volumes were measured along three orthogonal axes (a, b, and c) and calculated as abc/2 twice a week. The administration of drugs was done with Intraperitoneal injection. The tumor weight was determined at the end point. All animal experiments were approved by the Institutional Animal Care and Use Committee of Emory University.

### Statistical Analysis

Unless specified, data were plotted as mean ± SEM and experiments were performed at least twice. Statistical analyses were performed using GraphPad Prism (version 8, GraphPad Software), or R software (version 3.3.3). Student *t*‐test or one‐ or two‐way ANOVA, followed by Tukey multiple comparisons test was applied for statistical tests. Log‐rank (Mantel–Cox) test was applied for the comparison of survival curves. Pearson correlation or Spearman correlation was applied for the correlation analysis. *p* value < 0.05 was considered significant.

## Conflict of Interest

The authors declare no conflict of interest.

## Author Contributions

L.Z. and Y.Z. contributed equally to this work. Y.W., L.Z., and Y.Z. conceived the project. Y.W. supervised research. L.Z., Y.Z., X.C., J.C., A.U., X.S., Z.Z., and M.D. collected, analyzed, and interpreted data. Y.Z. performed bioinformatic and computational analyses. L.Z., Y.Z., and A.U. produced figures with input from Y.W., M.C., and K.K. oversaw clinical bioinformatic analyses. L.Z., Y.Z., and Y.W. prepared the manuscript. All authors discussed the results and commented on the manuscript.

## Supporting information

Supporting Information

## Data Availability

The data that support the findings of this study are available from the corresponding author upon reasonable request.
