# Supplementary material for: Tuning Immune‐Cold Tumor by Suppressing USP10/B7‐H4 Proteolytic Axis Reinvigorates Therapeutic Efficacy of ADCs
Source: Adv Sci (Weinh). 2024 Aug 29;11(40):2400757. doi: 10.1002/advs.202400757 (PMC11516061; doi:10.1002/advs.202400757)
Supplement: Supplementary file 1 — Supporting Information [file ADVS-11-2400757-s001.pdf]

ADVANCED SCIENCE
Open Access

Supporting Information

for *Adv. Sci.*, DOI 10.1002/advs.202400757

Tuning Immune-Cold Tumor by Suppressing USP10/B7-H4 Proteolytic Axis Reinvigorates Therapeutic Efficacy of ADCs

*Lidan Zeng, Yueming Zhu, Xin Cui, Junlong Chi, Amad Uddin, Zhuan Zhou, Xinxin Song, Mingji Dai, Massimo Cristofanilli, Kevin Kalinsky and Yong Wan**

Tuning immune-cold tumor by suppressing USP10/B7-H4 proteolytic axis reinvigorates therapeutic efficacy of ADCs

Lidan Zeng[1,2†], Yueming Zhu[1,2†], Xin Cui[1,2], Junlong Chi[1,3], Amad Uddin[1,2], Zhuan Zhou[4], Xinxin Song[4], Mingji Dai[1,5], Massimo Cristofanilli[6], Kevin Kalinsky[2,7], and Yong Wan[1,2,7]*

Supplementary Figures 1-9

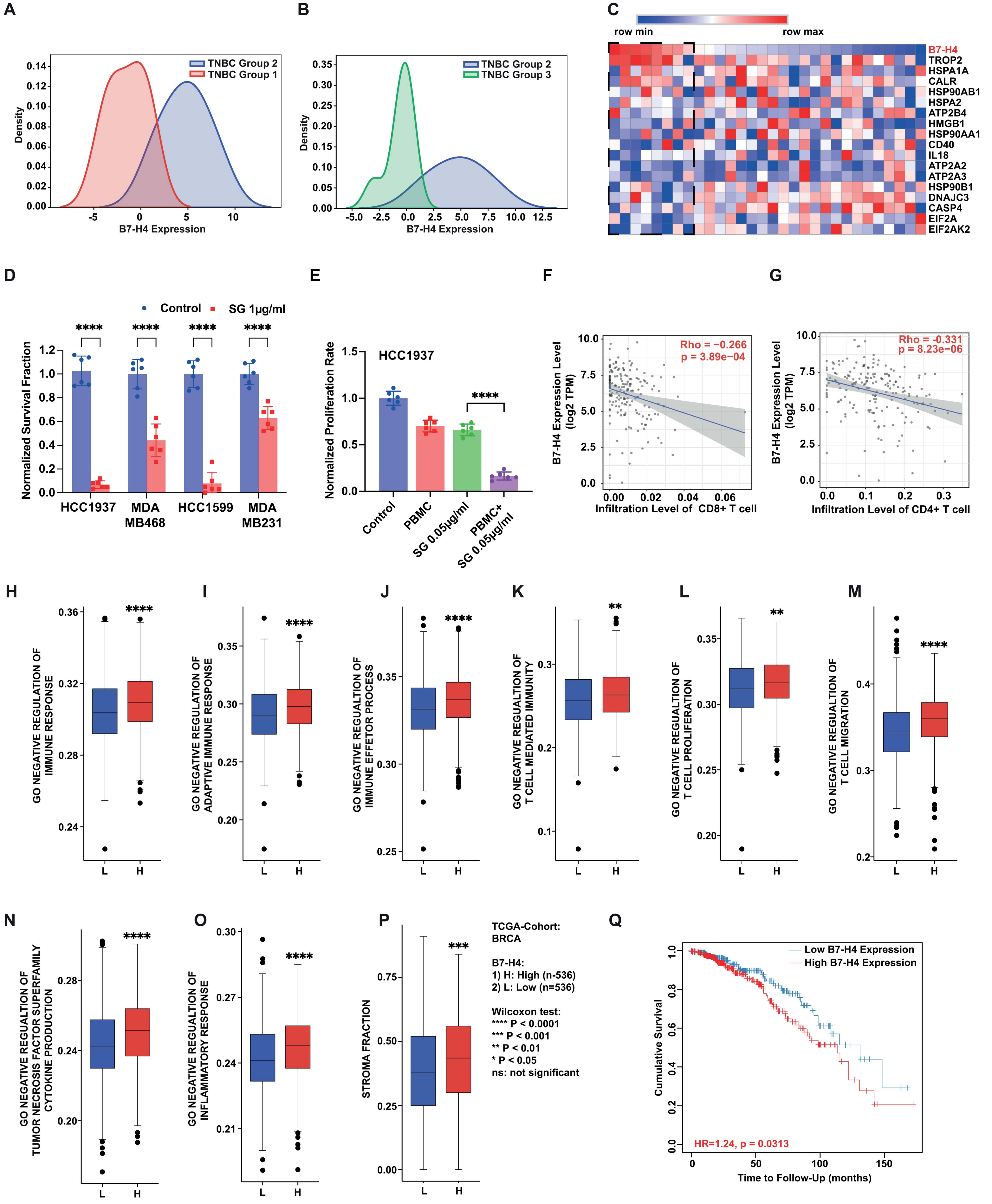

A
TNBC Group 2
TNBC Group 1
Density
B7-H4 Expression
B
TNBC Group 2
TNBC Group 3
Density
B7-H4 Expression
C
row min
row max
B7-H4
TROP2
HSPA1A
CALR
HSP90AB1
HSPA2
ATP2B4
HMGB1
HSP90AA1
CD40
IL18
ATP2A2
ATP2A3
HSP90B1
DNAJC3
CASP4
EIF2A
EIF2AK2
D
Control
SG 1μg/ml
Normalized Survival Fraction
HCC1937
MDA MB468
HCC1599
MDA MB231
E
HCC1937
Normalized Proliferation Rate
Control
PBMC
SG 0.05μg/ml
PBMC+ SG 0.05μg/ml
F
Rho = −0.266
p = 3.89e−04
B7-H4 Expression Level (log2 TPM)
Infiltration Level of CD8+ T cell
G
Rho = -0.331
p = 8.23e−06
B7-H4 Expression Level (log2 TPM)
Infiltration Level of CD4+ T cell
H
GO NEGATIVE REGULATION OF IMMUNE RESPONSE
I
GO NEGATIVE REGULATION OF ADAPTIVE IMMUNE RESPONSE
J
GO NEGATIVE REGUALTION OF IMMUNE EFFETOR PROCESS
K
GO NEGATIVE REGUALTION OF T CELL MEDIATED IMMUNITY
L
GO NEGATIVE REGUALTION OF T CELL PROLIFERATION
M
GO NEGATIVE REGUALTION OF T CELL MIGRATION
N
GO NEGATIVE REGUALTION OF TUMOR NECROSIS FACTOR SUPERFAMILY CYTOKINE PRODUCTION
O
GO NEGATIVE REGUALTION OF INFLAMMATORY RESPONSE
P
STROMA FRACTION
TCGA-Cohort: BRCA
B7-H4:
1) H: High (n-536)
2) L: Low (n=536)
Wilcoxon test:
**** P < 0.0001
*** P < 0.001
** P < 0.01
* P < 0.05
ns: not significant
Q
Low B7-H4 Expression
High B7-H4 Expression
Cumulative Survival
HR=1.24, p = 0.0313
Time to Follow-Up (months)

Figure S1

Figure S1. Elevated expression of B7-H4 in TNBC correlates with a poor tumor immunity. (A-B) B7-H4 expression density in three TNBC groups (A: G1 vs G2), (B: G2 vs G3) were calculated based on log2 relative protein expression. **(C)** Heatmap of B7-H4, ER stress and DAMP markers expression using CPTAC TNBC breast cancer cohort. Each column represents a sample; each row represents a protein. **(D)** HCC1937, MDA-MB-468, HCC1599 and MDA-MB-231 were treated with 1μg/ml SG for 48 hours, clonogenic survival were determined. **(E)** HCC1937 cells were cocultured with human PBMCs at Effector (E) to target (T) ratio (3:1) and treated with SG for 96 hours, cell proliferation rate was measured. **(F-G)** Spearman's correlation analysis showing the negative correlation between B7-H4 expression and the infiltration of CD8+ T cell (F) and CD4+ T cell (G). The correlation of B7-H4 with the immune infiltration level of CD8+ and CD4+ T cells in breast cancer patients (n=191) was analyzed using TIMER2.0 with tumor purity adjustment (http://timer.cistrome.org). a heatmap with the purity-adjusted spearman's rho is presented. **(H-P)** ssGSEA pathway analysis using breast cancer TCGA cohort showing high B7-H4 expression positively correlated with poor tumor immunity related pathways. **(Q)** Increased accumulation of B7-H4 levels correlates with poor outcomes in patients with breast cancer overall survival with Kaplan-Meier plotting. $^{**}p < 0.01$, $^{***}p < 0.001$, and $^{****}p < 0.0001$. Data (mean ± SEM) are representative of at least three independent experiments.

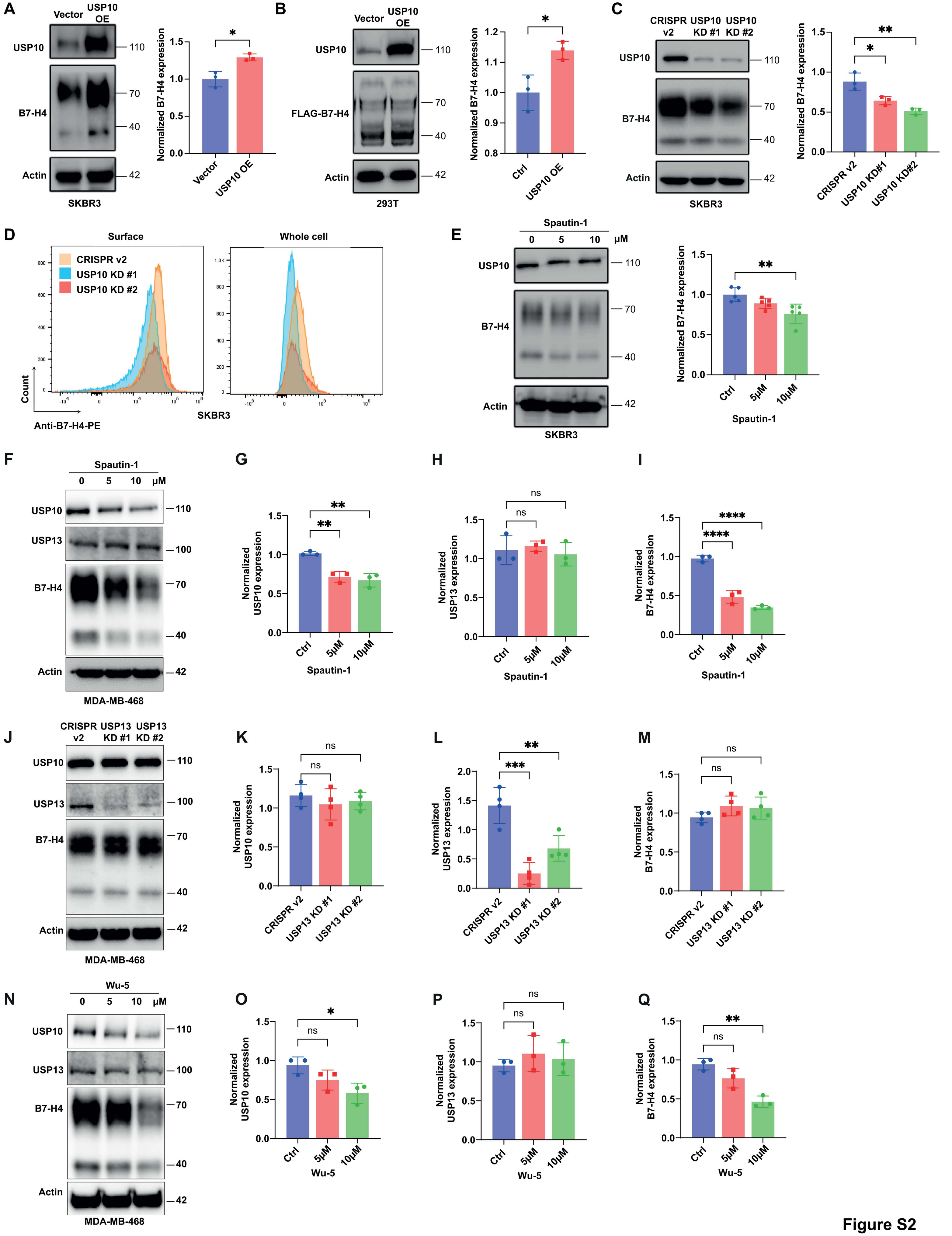
A
Vector
USP10 OE
USP10
B7-H4
Actin
110
70
40
42
SKBR3
Normalized B7-H4 expression
B
Vector
USP10 OE
USP10
FLAG-B7-H4
Actin
293T
Ctrl
C
CRISPR v2
USP10 KD #1
USP10 KD #2
SKBR3
USP10 KD#1
USP10 KD#2
D
Surface
Whole cell
CRISPR v2
USP10 KD #1
USP10 KD #2
Count
Anti-B7-H4-PE
SKBR3
E
Spautin-1
0
5
10
μM
5μM
10μM
F
MDA-MB-468
USP13
G
Normalized USP10 expression
H
Normalized USP13 expression
I
J
USP13 KD #1
USP13 KD #2
K
L
M
N
Wu-5
O
P
Q
ns

Figure S2

Figure S2. USP10 Regulates B7-H4 Expression and Stability.

(A) Immunoblotting analysis showing increased endogenous B7-H4 levels in SKBR3 cells with USP10 overexpression. **(B)** Immunoblotting analysis revealing upregulated exogenous FLAG-B7-H4 in 293T cells stably transduced with FLAG-B7-H4 expressing lentivirus upon USP10 overexpression. **(C)** USP10 knockdown decreases B7-H4 protein levels in SKBR3 cells, as shown by western blot analysis (left panel) and its quantification relative to Actin (right panel). **(D)** Decreased membrane and total B7-H4 in USP10 knockdown cells. SKBR3 cells were stained with PE conjugated anti-human B7-H4 antibody without or with permeabilization for surface B7-H4 and total B7-H4 respectively and subjected to flow cytometry analysis. **(E)** Immunoblotting analysis of USP10 and B7-H4 in SKBR3 cells post 24-hour treatment with the USP10 inhibitor, Spautin-1, indicating reduced B7-H4 expression. **(F-I)** MDA-MB-468 cells were treated with Spautin-1 at the indicated concentration for 48 hours and USP10, USP13 and B7H4 expression were determined by immunoblotting and quantified. **(J-M)** MDA-MB-468 with USP13 knockdown cell lines were established using different sgRNAs with the CRISPR/Cas9 system. An empty vector CRISPRv2 was used as the control. USP10, USP13 and B7H4 expression were determined by immunoblotting and quantified. **(N-Q)** MDA-MB-468 cells were treated with Wu-5 for 48 hours, USP10, USP13 and B7H4 expression were determined by immunoblotting and quantified. *p < 0.05, **p < 0.01, ***p < 0.001, ****p < 0.0001, and "ns" indicates no significant difference as determined by one way ANOVA test, Data (mean ± SEM) are representative of 3 independent experiments.

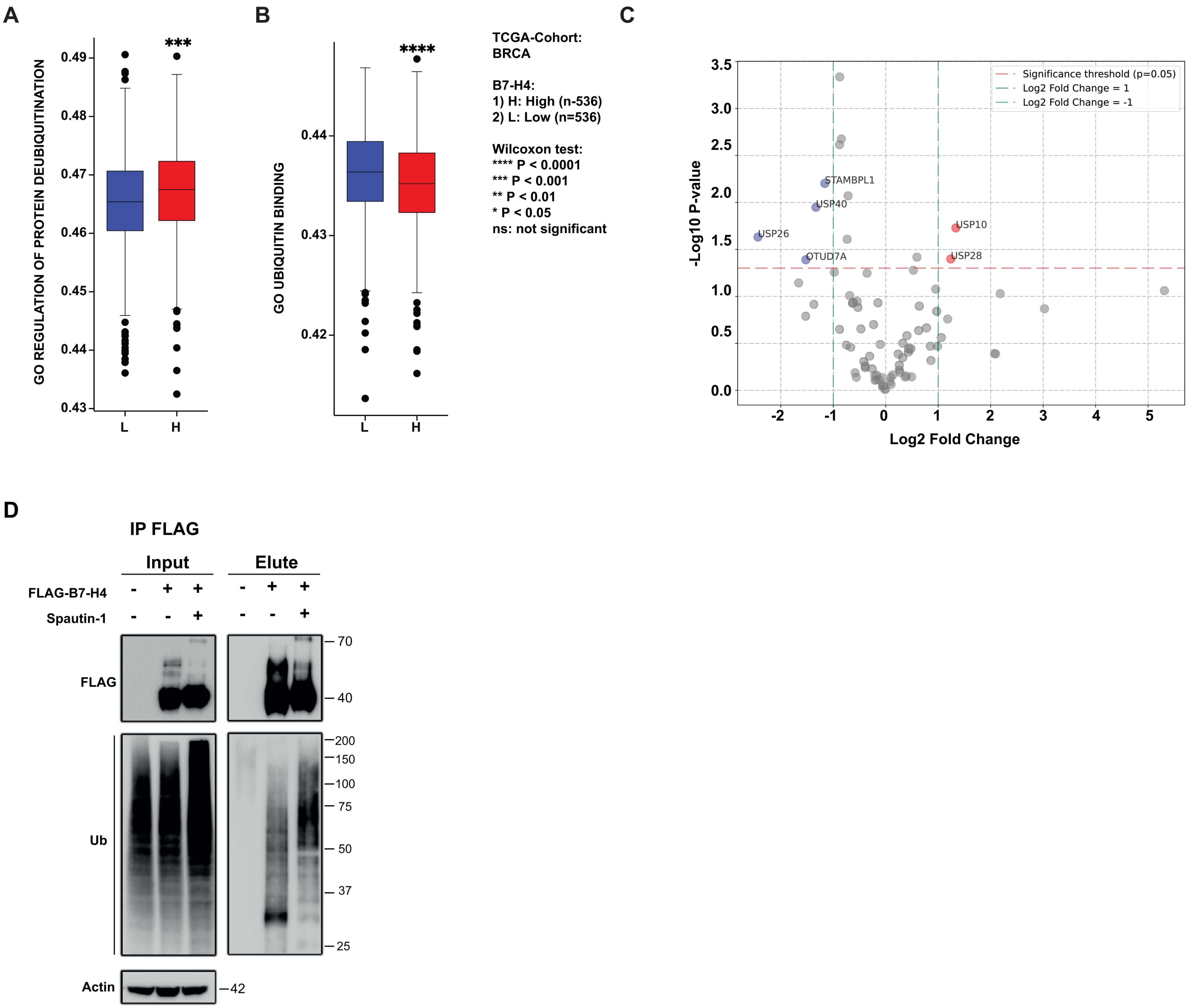
A
GO REGULATION OF PROTEIN DEUBIQUITINATION
***
L
H
B
GO UBIQUITIN BINDING
****
TCGA-Cohort: BRCA
B7-H4:
1) H: High (n-536)
2) L: Low (n=536)
Wilcoxon test:
**** P < 0.0001
*** P < 0.001
** P < 0.01
* P < 0.05
ns: not significant
C
Significance threshold (p=0.05)
Log2 Fold Change = 1
Log2 Fold Change = -1
STAMBPL1
USP40
USP26
OTUD7A
USP10
USP28
-Log10 P-value
Log2 Fold Change
D
IP FLAG
Input
Elute
FLAG-B7-H4
Spautin-1
FLAG
Ub
Actin

Figure S3

Figure S3. B7-H4 expression is correlated with ubiquitin-proteasome pathway.

(A-B) ssGSEA pathway analysis using breast cancer TCGA cohort showing high B7-H4 expression positively correlated with increased deubiquitylation activities (A) and decreased ubiquitin binding (B). **(C)** Volcano plots showing USP10 is upregulated in a subgroup of TNBC patient cohorts. Each circle represents one protein. The log fold change is represented on the x-axis. The y-axis shows the log10 of the p value. A p value of 0.05 and a fold change of 1 are indicated by red and green lines. ***$p < 0.001$, ****$p < 0.001$ by Wilcoxon test and data represent mean ± SEM. **(D)** HEK-293T stable cell line expressing FLAG-B7-H4 was treated in the presence or absence of 10μM spautin-1 for 24 hours. Then FLAG-B7-H4 was immunoprecipitated followed by immunoblotting using antibody against ubiquitin.

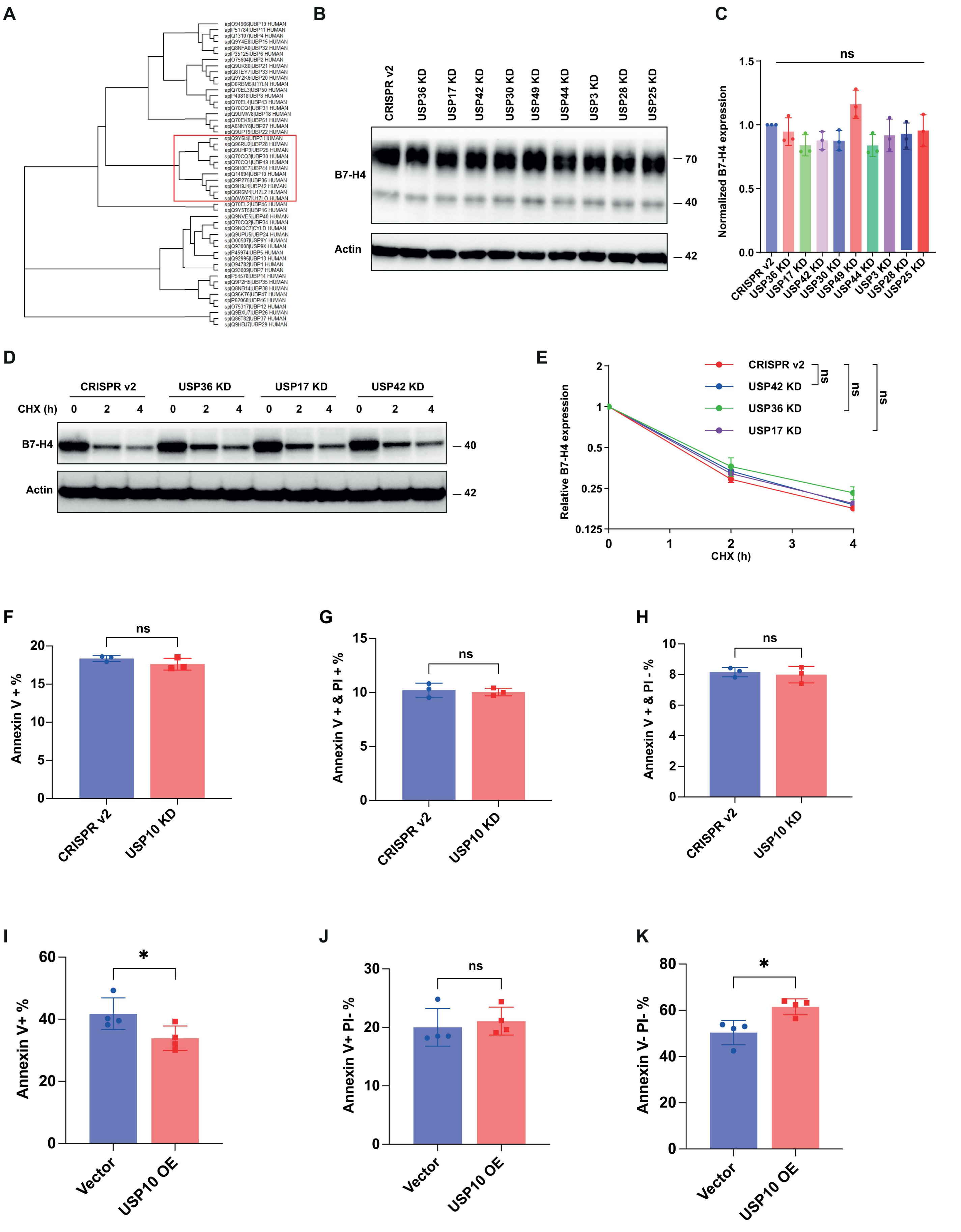
A
B
C
D
E
F
G
H
I
J
K
CRISPR v2
USP36 KD
USP17 KD
USP42 KD
USP30 KD
USP49 KD
USP44 KD
USP3 KD
USP28 KD
USP25 KD
B7-H4
Actin
70
40
42
ns
Normalized B7-H4 expression
CHX (h)
Relative B7-H4 expression
USP10 KD
Vector
USP10 OE
Annexin V + %
Annexin V + & PI + %
Annexin V + & PI - %
Annexin V+ %
Annexin V+ PI- %
Annexin V- PI- %
*

Figure S4

Figure S4. USP10 modulates B7-H4 stability and T cell function in MDA-MB-468 Cells.

(A) The phylogenetic tree of human USPs. The protein sequences of USPs were aligned, and the phylogenetic tree was generated. The branch containing USP10 (UBP10) is highlighted with a red rectangle. **(B-C)** MDA-MB-468 cells with different USP knockdown stable cell lines were established using different sgRNAs with the CRISPR/Cas9 system. An empty vector CRISPRv2 was used as the control. B7-H4 protein expression were determined. The knockdown of USPs in the same subfamily as USP10 does not reduce B7-H4 expression. (B) Representative images of western blot analysis. (C) Quantified intensity of B7-H4 expression from western blots. **(D-E)** B7-H4 turnover in different USPs knockdown stable MDA-MB-468 cell lines. (D) Pulse-chase B7-H4 turnover analysis for the control CRISPR v2 and USP knockdown stable MDA-MB-468 cells with 100 μg/mL cycloheximide. (E) Quantification of the amount of B7-H4 (40 kDa) normalized to the initial time point. **(F-H)** Annexin V/Propidium Iodide apoptosis assay analyzed by flow cytometry, demonstrating that knockdown of USP10 alone does not influence apoptosis in the absence of human PBMCs. **(I-K)** Flow cytometry images depicting the apoptotic profiles of MDA-MB-468 cells overexpressing USP10 in the presence of activated human PBMCs. Quantitative analysis of all (I) and early apoptotic events (J) and live events (K) in cancer cells upon USP10 overexpression during PBMC coculture. *$p < 0.05$, **$p < 0.01$, ***$p < 0.001$, ****$p < 0.0001$, and "ns" indicates no significant difference as determined by one-way ANOVA test. Data (mean ± SEM) are representative of at least three independent experiments.

A B

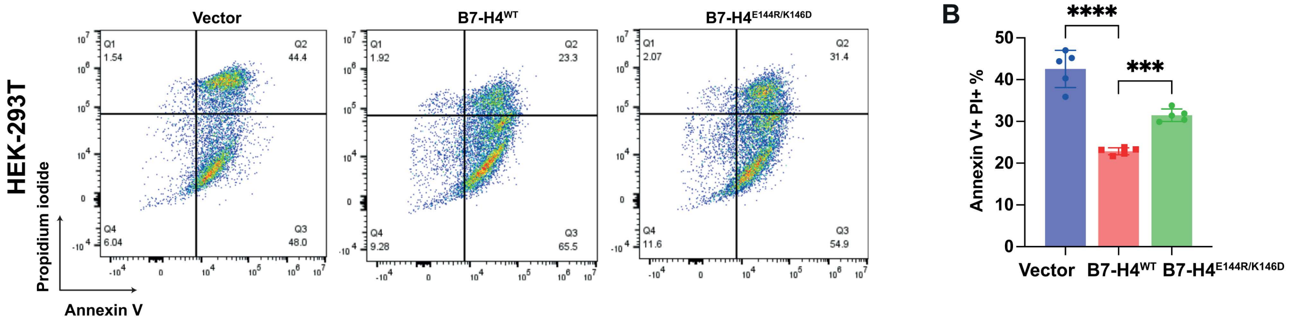
HEK-293T
Vector
B7-H4WT
B7-H4E144R/K146D
Q1 1.54
Q2 44.4
Q4 6.04
Q3 48.0
Q1 1.92
Q2 23.3
Q4 9.28
Q3 65.5
Q1 2.07
Q2 31.4
Q4 11.6
Q3 54.9
Propidium iodide
Annexin V
Annexin V+ PI+ %
****
***
Vector
B7-H4WT
B7-H4E144R/K146D

Figure S5

Figure S5. Reduced apoptosis in HEK-293T harboring B7-H4$^{E144R/K146D}$ mutation.

(A-B) HEK-293T cells expressing either B7-H4^{WT} and B7-H4$^{E144R/K146D}$ mutant were incubated with activated human PBMCs (A). Quantitative data show the percentage of cells in late (Annexin V+ and PI+) apoptotic stage (B). ***p< 0.001, and ****p < 0.0001. Data (mean ± SEM) are representative of at least three independent experiments.

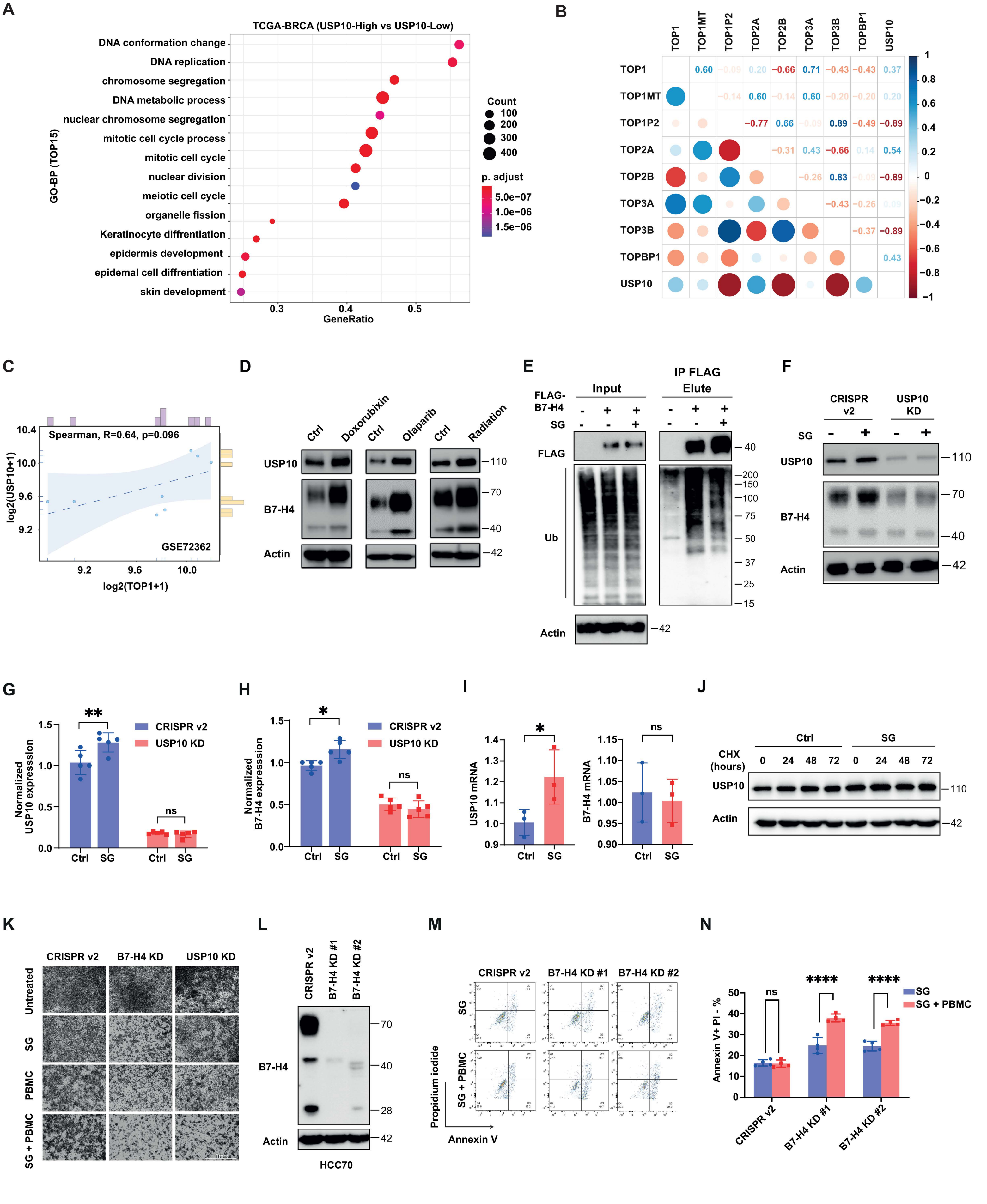

A
TCGA-BRCA (USP10-High vs USP10-Low)
DNA conformation change
DNA replication
chromosome segregation
DNA metabolic process
nuclear chromosome segregation
mitotic cell cycle process
mitotic cell cycle
nuclear division
meiotic cell cycle
organelle fission
Keratinocyte diffrentiation
epidermis development
epidemal cell diffrentiation
skin development
GO-BP (TOP15)
GeneRatio
Count
p. adjust
B
TOP1 TOP1MT TOP1P2 TOP2A TOP2B TOP3A TOP3B TOPBP1 USP10
C
Spearman, R=0.64, p=0.096
GSE72362
log2(USP10+1)
log2(TOP1+1)
D
Ctrl Doxorubixin Ctrl Olaparib Ctrl Radiation
USP10
B7-H4
Actin
E
Input
IP FLAG Elute
FLAG-B7-H4
SG
FLAG
Ub
Actin
F
CRISPR v2
USP10 KD
SG
USP10
B7-H4
Actin
G
CRISPR v2
USP10 KD
Normalized USP10 expression
H
Normalized B7-H4 expression
I
USP10 mRNA
B7-H4 mRNA
J
Ctrl SG
CHX (hours)
USP10
Actin
K
CRISPR v2 B7-H4 KD USP10 KD
Untreated
SG
PBMC
SG + PBMC
L
CRISPR v2
B7-H4 KD #1
B7-H4 KD #2
B7-H4
Actin
HCC70
M
CRISPR v2 B7-H4 KD #1 B7-H4 KD #2
SG
SG + PBMC
Propidium iodide
Annexin V
N
SG
SG + PBMC
Annexin V+ PI - %
CRISPR v2
B7-H4 KD #1
B7-H4 KD #2

Figure S6

Figure S6. SG treatment stabilizes B7-H4 by upregulating USP10.

(A) Go-BP pathway analysis using TCGA dataset identifying the most affected pathways between USP10 high vs USP10 low breast cancer groups. **(B-C)** Spearman's rank gene correlation analysis showing the USP10 expression is highly positively correlated with DNA topoisomerases including SG payload target TOP1 (C) using GSE39042 database. **(D)** MDA-MB468 cells were treated with 0.5μM doxorubicin, 5μM Olaparib and 2Gy radiation. USP10 and B7-H4 protein levels were determined by immunoblotting. **(E)** MDA-MB-468 cells stably expressing FLAG-B7-H4 were treated with 100 ng/mL SG or left untreated for 24 hours. Subsequently, FLAG-B7-H4 was immunoprecipitated and its ubiquitylation level was analyzed. **(F)** Representative immunoblots show USP10 and B7-H4 levels in cells with CRISPR v2 and USP10 knockdown, treated with or without 100 ng/mL SG. **(G)** Quantification of B7-H4 protein intensity. **(H)** Quantification of USP10 protein intensity. $^{*}p < 0.05$, $^{**}p < 0.01$ by the one-way ANOVA test. Data (mean ± SEM) are representative of at least three independent experiments. **(I)** Quantification mRNA levels of USP10 normalized with β-actin with qPCR in MDA-MB-468 in the presence or absence of 100ng/mL SG. $^{*}p < 0.05$ by the student- test, Data (mean ± SEM) are representative of at least three independent experiments. **(J)** Analysis of protein turnover for USP10 in MDA-MB-468 cells using pulse-chase analysis with 100 ng/mL cycloheximide in the presence or absence of 100 ng/mL SG. **(K)** Representative images of MDA-MB-468-B7-H4 KD and MDA-MB-468-USP10 KD cells cocultured with human PBMCs at Effector (E) to target (T) ratio (3:1) and treated with SG (0.1 μg/ml). **(L)** Immunoblotting confirming B7-H4 knockdown in HCC70 cells using CRISPR/Cas9. Two different sgRNAs were utilized to establish B7-H4 knockdown in HCC70 cell lines, with an empty CRISPRv2 vector serving as the control. **(M-N)** Flow cytometry analysis of HCC70 cells co-cultured with PBMCs demonstrates increased apoptosis in B7-H4 knockdown cells under SG and PBMC combination treatment. (M) Representative flow cytometry results. (N) Quantitative analysis of the percentage of early apoptosis (Annexin V+ and PI-) in cancer cells. $^{****}p < 0.0001$ by the two-way ANOVA. Data (mean ± SEM) are representative of at least three independent experiments.

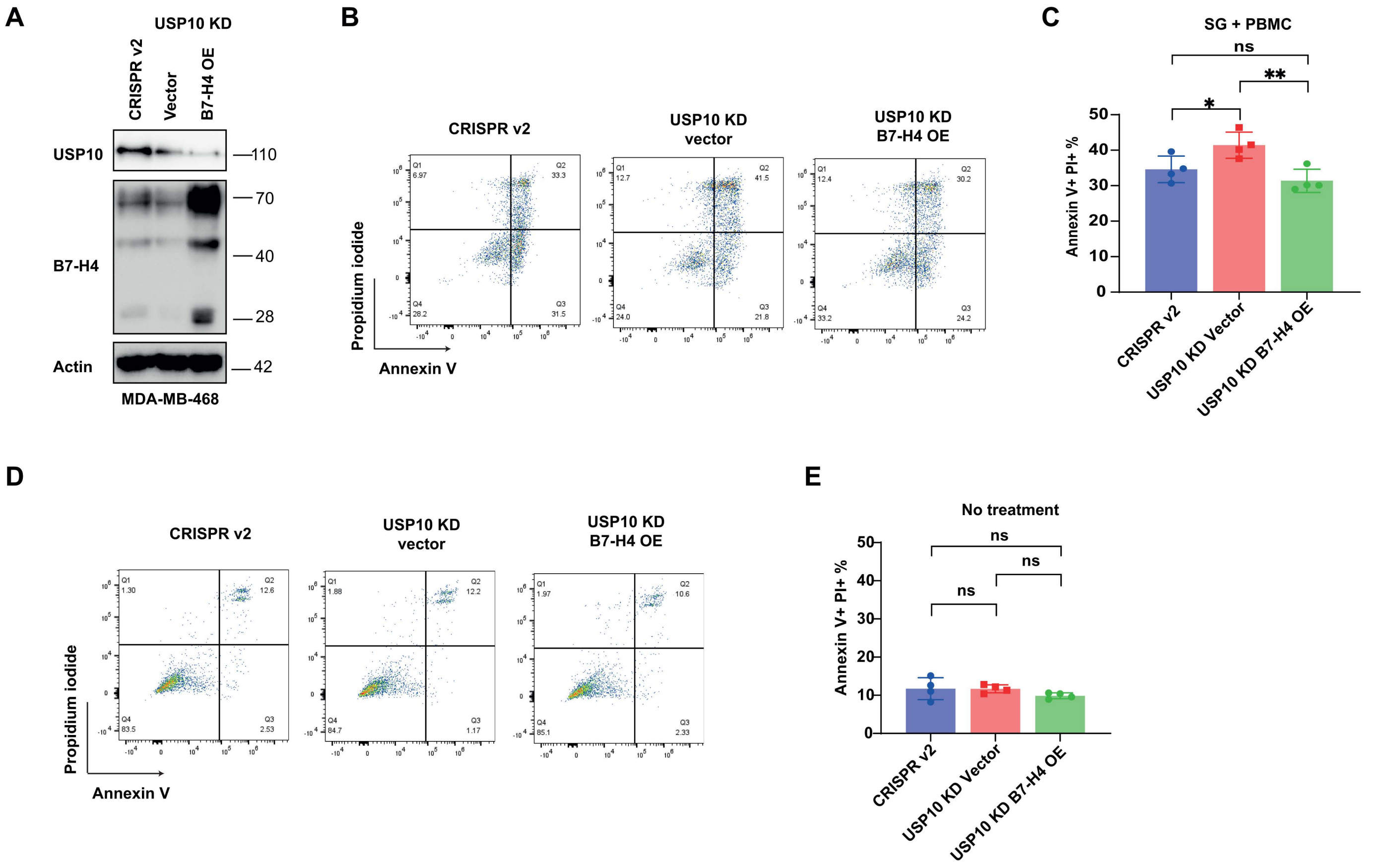
A
USP10 KD
CRISPR v2
Vector
B7-H4 OE
USP10
110
70
40
28
B7-H4
Actin
42
MDA-MB-468
B
CRISPR v2
USP10 KD
vector
USP10 KD
B7-H4 OE
Propidium iodide
Annexin V
C
SG + PBMC
ns
**
*
Annexin V+ PI+ %
50
40
30
20
10
0
CRISPR v2
USP10 KD Vector
USP10 KD B7-H4 OE
D
CRISPR v2
USP10 KD
vector
USP10 KD
B7-H4 OE
Propidium iodide
Annexin V
E
No treatment
ns
ns
ns
Annexin V+ PI+ %
50
40
30
20
10
0
CRISPR v2
USP10 KD Vector
USP10 KD B7-H4 OE

Figure S7

Figure S7. Reintroduction of B7-H4 promotes resistance to SG in TNBC cells

(A) Restoration of B7-H4 in USP10 KD MDA-MB-468 stable cell lines via lentiviral transduction. Representative immunoblots of USP10 and B7-H4 confirm B7-H4 restoration in these cell lines. **(B-C)** Flow cytometry analysis of MDA-MB-468 cell lines cocultured with PBMCs reveals that restoration of B7-H4 completely rescues the effects of USP10 KD under PBMC and SG (100ng/mL) treatment. (B) Representative flow cytometry images; (C) Quantitative analysis showing the percentage of late apoptotic (Annexin V+ and PI+) cancer cells. **(D-E)** Annexin V/Propidium Iodide apoptosis assays by flow cytometry indicate that knockdown of USP10 alone or with restored B7-H4 does not affect apoptosis under normal culture conditions or with SG (100ng/mL) treatment. (D) Representative results; (E) Quantitative analysis of late apoptotic cells. $*p < 0.05$, $**p < 0.01$, and "ns" indicates no significant difference by the two-way ANOVA test, Data (mean ± SEM) are representative of at least three independent experiments.

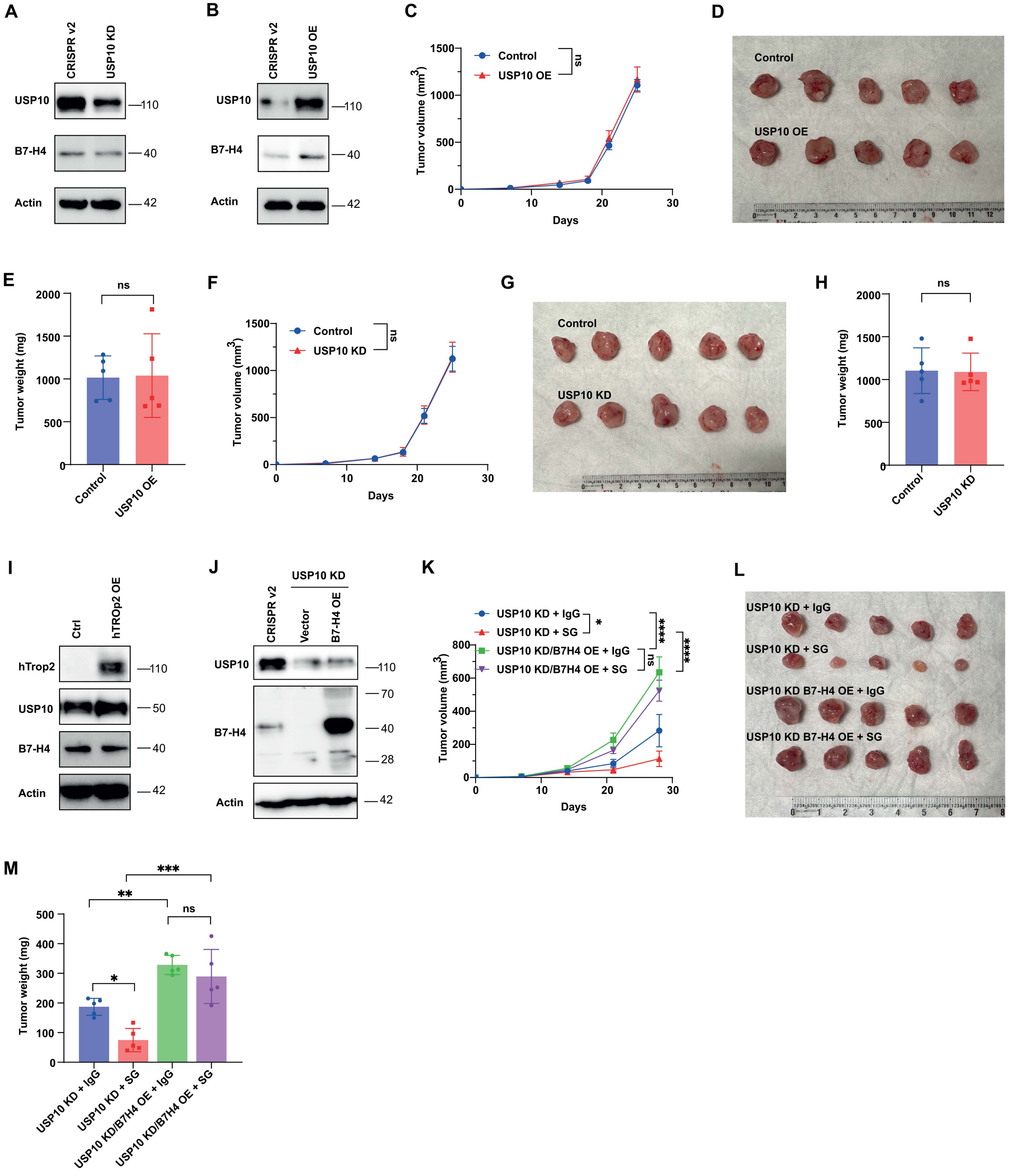
A
CRISPR v2
USP10 KD
USP10
110
B7-H4
40
Actin
42
B
CRISPR v2
USP10 OE
USP10
110
B7-H4
40
Actin
42
C
Control
USP10 OE
ns
Tumor volume (mm3)
Days
D
Control
USP10 OE
E
ns
Tumor weight (mg)
Control
USP10 OE
F
Control
USP10 KD
ns
Tumor volume (mm3)
Days
G
Control
USP10 KD
H
ns
Tumor weight (mg)
Control
USP10 KD
I
Ctrl
hTROp2 OE
hTrop2
110
USP10
50
B7-H4
40
Actin
42
J
USP10 KD
CRISPR v2
Vector
B7-H4 OE
USP10
110
B7-H4
70
40
28
Actin
42
K
USP10 KD + IgG
USP10 KD + SG
USP10 KD/B7H4 OE + IgG
USP10 KD/B7H4 OE + SG
*
ns
****
****
Tumor volume (mm3)
Days
L
USP10 KD + IgG
USP10 KD + SG
USP10 KD B7-H4 OE + IgG
USP10 KD B7-H4 OE + SG
M
***
**
ns
*
Tumor weight (mg)
USP10 KD + IgG
USP10 KD + SG
USP10 KD/B7H4 OE + IgG
USP10 KD/B7H4 OE + SG

Figure S8

Figure S8. Reintroduction of B7-H4 in 4T1-USP10 knockdown murine model promotes tumor growth and SG resistance

(A-B) Representative immunoblotting images of mUSP10 and mB7-H4 across various 4T1 stable cell lines. **(C-H)** 4T1 control and 4T1-mUSP10 overexpression (C-E) or 4T1-mUSP10 knockdown (F-H) breast cancer cells were orthotopically injected into the right fourth mammary gland of the nude mice. Tumor growth curve was plotted (C, F) and tumor was harvested at 25 days after tumor challenge, weight was imaged and measured (D-E, G-H). **(I)** Representative immunoblotting images of the control and 4T1-hTrop2 stable cell lines. **(J)** Restoration of mB7-H4 in mUSP10 KD 4T1-hTrop2 stable cell lines via lentiviral transduction. Immunoblotting confirms the levels of mB7-H4 and mUSP10. **(K)** Tumor growth curve of 4T1-hTROP2 mUSP10 KD and 4T1-hTROP2 mUSP10 KD mB7-H4 OE cells, which were orthotopically injected into the left fourth mammary fat pad and allowed to grow around 100 mm3, followed by injection of spautin-1 (20 mg/kg, i.p.) for two times/week and SG (10 mg/kg, i.p.) for 3 times. The tumor growth was monitored twice per week. **(L-M)** Tumor was harvested at 28 days after tumor challenge, imaged (L) and weighted (M). *$p < 0.05$, **$p < 0.01$, ***$p < 0.001$, ****$p < 0.0001$ and "ns" indicates no significant difference by the one way or two-way ANOVA test, Data (mean ± SEM) are representative of at least two independent experiments with five to ten independently analyzed mice per group.

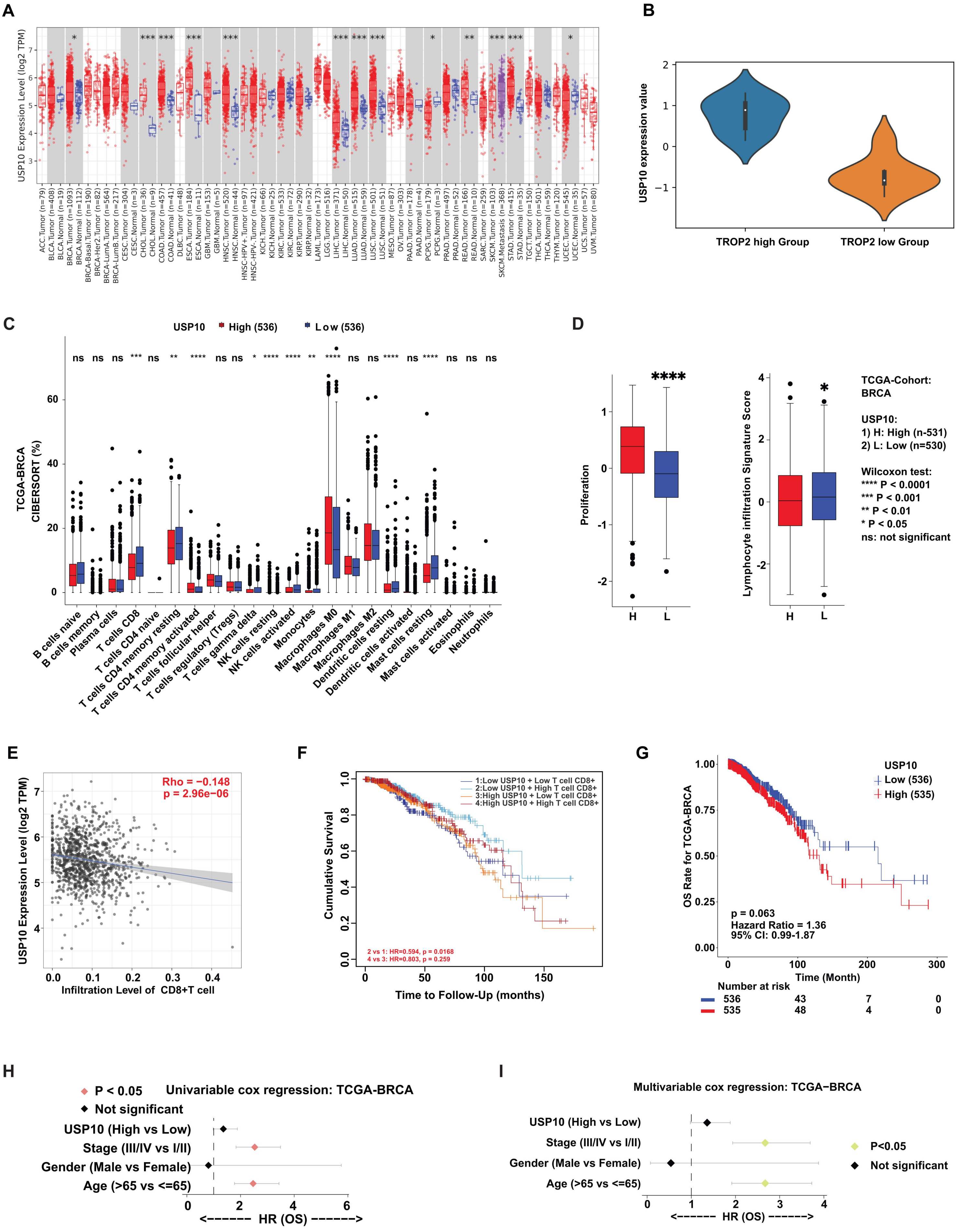

A
USP10 Expression Level (log2 TPM)
ACC.Tumor (n=79)
BLCA.Tumor (n=408)
BLCA.Normal (n=19)
BRCA.Tumor (n=1093)
BRCA.Normal (n=112)
BRCA-Basal.Tumor (n=190)
BRCA-Her2.Tumor (n=82)
BRCA-LumA.Tumor (n=564)
BRCA-LumB.Tumor (n=217)
CESC.Tumor (n=304)
CESC.Normal (n=3)
CHOL.Tumor (n=36)
CHOL.Normal (n=9)
COAD.Tumor (n=457)
COAD.Normal (n=41)
DLBC.Tumor (n=48)
ESCA.Tumor (n=184)
ESCA.Normal (n=11)
GBM.Tumor (n=153)
GBM.Normal (n=5)
HNSC.Tumor (n=520)
HNSC.Normal (n=44)
HNSC-HPV+.Tumor (n=97)
HNSC-HPV-.Tumor (n=421)
KICH.Tumor (n=66)
KICH.Normal (n=25)
KIRC.Tumor (n=533)
KIRC.Normal (n=72)
KIRP.Tumor (n=290)
KIRP.Normal (n=32)
LAML.Tumor (n=173)
LGG.Tumor (n=516)
LIHC.Tumor (n=371)
LIHC.Normal (n=50)
LUAD.Tumor (n=515)
LUAD.Normal (n=59)
LUSC.Tumor (n=501)
LUSC.Normal (n=51)
MESO.Tumor (n=87)
OV.Tumor (n=303)
PAAD.Tumor (n=178)
PAAD.Normal (n=4)
PCPG.Tumor (n=179)
PCPG.Normal (n=3)
PRAD.Tumor (n=497)
PRAD.Normal (n=52)
READ.Tumor (n=166)
READ.Normal (n=10)
SARC.Tumor (n=259)
SKCM.Tumor (n=103)
SKCM.Metastasis (n=368)
STAD.Tumor (n=415)
STAD.Normal (n=35)
TGCT.Tumor (n=150)
THCA.Tumor (n=501)
THCA.Normal (n=59)
THYM.Tumor (n=120)
UCEC.Tumor (n=545)
UCEC.Normal (n=35)
UCS.Tumor (n=57)
UVM.Tumor (n=80)
B
USP10 expression value
TROP2 high Group
TROP2 low Group
C
USP10
High (536)
Low (536)
TCGA-BRCA CIBERSORT (%)
B cells naive
B cells memory
Plasma cells
T cells CD8
T cells CD4 naive
T cells CD4 memory resting
T cells CD4 memory activated
T cells follicular helper
T cells regulatory (Tregs)
T cells gamma delta
NK cells resting
NK cells activated
Monocytes
Macrophages M0
Macrophages M1
Macrophages M2
Dendritic cells resting
Dendritic cells activated
Mast cells resting
Mast cells activated
Eosinophils
Neutrophils
D
Proliferation
Lymphocyte infiltration Signature Score
TCGA-Cohort: BRCA
USP10:
1) H: High (n-531)
2) L: Low (n=530)
Wilcoxon test:
**** P < 0.0001
*** P < 0.001
** P < 0.01
* P < 0.05
ns: not significant
E
Rho = −0.148
p = 2.96e−06
USP10 Expression Level (log2 TPM)
Infiltration Level of CD8+T cell
F
1:Low USP10 + Low T cell CD8+
2:Low USP10 + High T cell CD8+
3:High USP10 + Low T cell CD8+
4:High USP10 + High T cell CD8+
Cumulative Survival
2 vs 1: HR=0.594, p = 0.0168
4 vs 3: HR=0.803, p = 0.259
Time to Follow-Up (months)
G
USP10
Low (536)
High (535)
OS Rate for TCGA-BRCA
p = 0.063
Hazard Ratio = 1.36
95% CI: 0.99-1.87
Time (Month)
Number at risk
536 43 7 0
535 48 4 0
H
P < 0.05
Univariable cox regression: TCGA-BRCA
Not significant
USP10 (High vs Low)
Stage (III/IV vs I/II)
Gender (Male vs Female)
Age (>65 vs <=65)
<------ HR (OS) ------>
I
Multivariable cox regression: TCGA−BRCA
USP10 (High vs Low)
Stage (III/IV vs I/II)
Gender (Male vs Female)
Age (>65 vs <=65)
P<0.05
Not significant
<------ HR (OS) ------>

Figure S9

Figure S9. Increased USP10 expression in TNBC correlated with poor tumor immunity.

(A) Differential gene expression analysis between tumor and adjacent normal tissues for any gene of interest across all TCGA tumors. USP10 expression is significantly upregulated in breast cancer. **(B)** USP10 expression levels in two groups of TNBCs, categorized as TROP2-high and TROP2-low, were analyzed and presented using log2 relative protein expression values. **(C)** CIBERSORT analysis using TCGA-BRCA dataset showing that high USP10 levels correlate with decreased $CD8^{+}$ T cell populations and an immunosuppressive phenotype. **(D)** ssGSEA pathway analysis using breast cancer TCGA cohort showing high USP expression positively correlated with tumor proliferation (left panel) and decreased lymphocyte infiltration (right panel). **(E)** Spearman's correlation analysis showing the negative correlation between USP10 expression and CD8 T cell infiltration. **(F)** Kaplan-Meier curves using multivariable Cox proportional hazard model for the corresponding $CD8^{+}$ T cells and B7-H4 expression. The infiltration and expression level are divided into low and high levels. **(G)** Kaplan-Meier plotting showing that increased USP10 levels associated with poor overall survival in patients with breast cancer. **(H)** Univariable cox regression analysis showing high USP10 expression as an increased survival hazard in breast cancer patient overall survival. **(I)** USP10 was identified as a survival hazard using multivariable Cox proportional hazard model. $*p < 0.05$, $**p < 0.01$, $***p < 0.001$, and $****p < 0.0001$ by Wilcoxon test and data represent mean ± SEM.
